# Novel Lipids to Regulate Obesity and Brain Function: Comparing Available Evidence and Insights from QSAR In Silico Models

**DOI:** 10.3390/foods12132576

**Published:** 2023-07-01

**Authors:** Francisca S. Teixeira, Paula T. Costa, Ana M. S. Soares, Ana Luiza Fontes, Manuela E. Pintado, Susana S. M. P. Vidigal, Lígia L. Pimentel, Luís M. Rodríguez-Alcalá

**Affiliations:** CBQF—Centro de Biotecnologia e Química Fina—Laboratório Associado, Escola Superior de Biotecnologia, Universidade Católica Portuguesa, Rua Diogo Botelho 1327, 4169-005 Porto, Portugal; fsteixeira@ucp.pt (F.S.T.); ptcosta@ucp.pt (P.T.C.); amsoares@ucp.pt (A.M.S.S.); afontes@ucp.pt (A.L.F.); mpintado@ucp.pt (M.E.P.); svidigal@ucp.pt (S.S.M.P.V.); lpimentel@ucp.pt (L.L.P.)

**Keywords:** ergosterol, policosanol, sphingomyelin, krill phospholipids, obesity, neurological disorders, ADMET, in silico QSAR

## Abstract

Lipid molecules, such as policosanol, ergosterol, sphingomyelin, omega 3 rich phosphatidylcholine, α-tocopherol, and sodium butyrate, have emerged as novel additions to the portfolio of bioactive lipids. In this state-of-the-art review, we discuss these lipids, and their activity against obesity and mental or neurological disorders, with a focus on their proposed cellular targets and the ways in which they produce their beneficial effects. Furthermore, this available information is compared with that provided by in silico Absorption, Distribution, Metabolism, Excretion, and Toxicity (ADMET) models in order to understand the usefulness of these tools for the discovery of new bioactive compounds. Accordingly, it was possible to highlight how these lipids interact with various cellular targets related to the molecule transportation and absorption (e.g., α-tocopherol transfer protein for α-Tocopherol, ATP-binding cassette ABC transporters or Apolipoprotein E for sphingomyelins and phospholipids) or other processes, such as the regulation of gene expression (involving Sterol Regulatory Element-Binding Proteins for ergosterol or Peroxisome Proliferator-Activated Receptors in the case of policosanol) and inflammation (the regulation of interleukins by sodium butyrate). When comparing the literature with in silico Quantitative Structure–Activity Relationship (QSAR) models, it was observed that although they are useful for selecting bioactive molecules when compared in batch, the information they provide does not coincide when assessed individually. Our review highlights the importance of considering a broad range of lipids as potential bioactives and the need for accurate prediction of ADMET parameters in the discovery of new biomolecules. The information presented here provides a useful resource for researchers interested in developing new strategies for the treatment of obesity and mental or neurological disorders.

## 1. Introduction

Lipids have been always associated with human health as these molecules were primarily considered as energy storage molecules, therefore as playing a role in body weight gain. However, recent advances in lipid research have led to a paradigm shift, revealing their diverse and crucial roles in the regulation of cellular pathways such as, for example, those related to cell survival and growth (e.g., phosphatidylinositol-3,4,5-trisphosphate, PIP3), inflammation (e.g., prostaglandins, leukotrienes, and thromboxanes) or stress response (e.g., steroids hormones as cortisol). This new understanding has generated a growing interest in the identification of novel lipids that could potentially target and modulate health conditions. As the global prevalence of obesity and mental and neurological disorders, such as dementia, continues to rise, there is an urgent need to explore innovative therapeutic approaches. This review delves into the potential of ergosterol, octacosanol, N-palmitoylsphingomyelin, α-tocopherol, phosphatidylcholine (16:0/20:5), phosphatidylcholine (16:0/22:6), butyrate, and sodium butyrate in addressing these challenges and compares existing evidence with insights from recent in silico Quantitative Structure–Activity Relationship (QSAR) models to support future research and therapeutic strategies.

## 2. Prevalence and Current Impact of Obesity and Dyslipidemia

The prevalence of non-communicable diseases, such as obesity and type II diabetes, has been increasing in the European region, affecting both adults and children. Obesity is usually defined by Body Mass Index (BMI) values equal or superior to 30 kg/m^2^, and when values range between 25 and 29.9 kg/m^2^ it is classified as overweight [1]. In fact, one in three children and/or adolescents is overweight or obese and the mean BMI reached higher levels during the COVID-19 pandemic [2].

Some emerging diseases that may be related to the rising obesity index are Non-Alcoholic Steatohepatitis (NASH) and Metabolic Syndrome (MetS) [3]. The most prevalent chronic liver disease worldwide is Non-Alcoholic Fatty Liver Disease (NAFLD), and its progressive phenotype is characterized as NASH [3]. Regarding NAFLD, it is estimated that the percentage of incidence in the adult European population is approximately 19–30%, a value that will increase in the next decades with the prevalence of obesity [2]. The broad-spectrum of MetS is associated with obesity and characterized by some abnormalities such as NAFLD and dyslipidemia, which is characterized by the imbalance of lipids (i.e., total cholesterol, Low-Density Lipoprotein [LDL] cholesterol, triglycerides [TGs], and High-Density Lipoprotein [HDL] cholesterol) [2,4]. Thus, the implementation of strategies and efforts to revert this situation is urgent.

A study involving 50 overweight and obese patients, with BMI levels between 25 and 33 kg/m^2^, revealed that, in these conditions, erythrocytes can be subjected to oxidative damage and present increased cholesterol content, lower concentrations of omega-3 fatty acids (FAs) and thus a decrease in membrane fluidity [5,6]. In obese patients, the increase in oxidative stress and cell damage is also related to mitochondrial dysfunction [7]. Chronic inflammation can be triggered by the metabolic imbalance resulting from obesity, since immune cells are stimulated to increase the production of some cytokines, such as tumor necrosis factor-α (TNF-α) [8].

The control of obesity passes through lifestyle management (e.g., eating behavior and physical activity), psychological approaches, pharmacotherapy, and, in the final instance, metabolic and bariatric surgery. Pharmacotherapy treatments with orlistat, liraglutide, and the combination of bupropion and naltrexone promote weight loss and received European Medicines Agency (EMA) approval [2]. However, studies elsewhere have described how antiobesity drugs can exert negative side effects [9]. Thus, Orlistat, a pancreatic lipase inhibitor, is considered a long-term weight management agent with known side effects such as abdominal pain and diarrhea. On the other hand, the short-term utilization of the appetite suppressant diethylpropion can affect blood pressure and provoke insomnia and headaches. Also, Liraglutide, used for weight loss during Type II Diabetes, can trigger nausea. The anti-depressant bupropion has shown modest weight loss, while naltrexone (i.e., opioid addiction and alcoholism treatment) is not associated with weight reduction. Nevertheless, their combination is known for its success in helping in weight control, although it is associated to nausea, headache, and insomnia. Consequently, clinical interventions should be used along with nutritional education aiming to promote healthier behaviors in the patients [1,4,9].

The control of hypercholesterolemia usually involves statins and ezetimibe drugs, but the cholesterol-lowering and hypolipidemia activity of some natural nutraceuticals containing stigmasterol, β-sitosterol, ergosterol, policosanol, and sphingomyelin are being studied worldwide [8,10,11,12,13].

### 2.1. Ergosterol

The bioactivity of phytosterols in functional foods (i.e., margarine and dairy foods) has gained increasing relevance according to clinical studies that confirmed its cholesterol-lowering properties [14]. Moreover, sterols are structural components of cells that contribute to membrane fluidity [15]. It is well known that some sterols are nutritional modulators of hypercholesterolemia, inflammatory processes, oxidative stress, and gut dysbiosis [7].

Cholesterol is the main sterol produced by mammals, while ergosterol is synthetized by fungi, and β-sitosterol or campesterol by plants [16]. Fungal sterols’ biosynthesis (i.e., ergosterol) involves several enzymes and squalene oxide as an intermediate in the isoprenoid pathway, which is analogous to mammal cholesterol biosynthesis via the acetate-mevalonate route [17]. Structurally, ergosterol differs from cholesterol as it contains double bonds in the B ring (between carbons 7–8) and on the side chain (carbons 22–23), as well as a methyl group on carbon 24.

Since the early decades of the 20th century, ergosterol has been described as a vitamin D-related substance [18]. Some authors hypothesize that, in humans, low plasma levels of vitamin D and its metabolites (i.e., calcidiol) can be connected to higher body fat accumulation and obesity prevalence [19]. Ergosterol (5,7-diene oxysterol or pro-vitamin D_2_) has a similar structure to ergocalciferol and cholecalciferol (commonly named vitamin D_2_ and vitamin D_3_, respectively) and is considered the primary sterol in fungal membrane cells, such as yeasts and mushrooms. In humans, vitamin D_3_ is produced from 7-dehydrocholesterol during skin exposure to ultraviolet (UV) radiation and a lack of vitamin D production makes its supplementation a crucial practice. In fact, intentional UV treatment of ergosterol from mushrooms and yeasts has been implemented to produce vitamin D_2_ for dietary supplements [20,21,22]. Ergosterol is considered a valuable molecule and its estimated cost is USD 800/100 g (98% of purity) [23].

Ultraviolet-B (UV-B) radiation applied to ergosterol obtained from white button mushroom powder has been proven as an effective procedure to obtain vitamin D_2_ (741.5 ± 23.7 μg/g dry weight) [24]. For instance, Gil et al. [25] extracted ergosterol from *Agaricus bisporus* mushroom powder through CO_2_ Supercritical Fluid Extraction, obtaining ergosterol at 37.3% of dry weight. In accordance with the previous results, lyophilized *Agaricus bisporus* wastes reached 36.72 ± 0.01 mg/g extract [23], and the hexane fraction from the same species reached 34.96 ± 0.47 mg/g extract [8]. Ergosterol was also found in maitake and shiitake mushrooms at 79.2 and 84.9 mg/100 g, respectively [21]. Furthermore, *Saccharomyces cerevisiae* produces ergosterol as a cellular antioxidant, especially in adverse conditions caused by perturbations induced by hydroperoxides in medium [15].

Research conducted on mice after ergosterol intake of 7 mg/kg for 6 weeks demonstrated a significant increase in serum, liver, and kidney concentrations of vitamin D_3_. Furthermore, liver cholesterol and TG contents did not vary with this intake [20].

The 3-hidroxi-3-methyl-glutaril-CoA (HMG-CoA) reductase is one of the most targeted enzymes involved in the cholesterol biosynthetic pathway. Statins inhibit HMG-CoA reductase, which reduces the hepatic synthesis of cholesterol. Natural products targeting the downregulation of this enzyme and, consequently, exerting hypocholesterolemic activity, are currently being studied [26].

Lipogenesis is regulated by some known enzymes such as Fatty Acid Synthase (FAS) and HMG-CoA reductase. Thus, hepatic cholesterol clearance and maintenance of plasma levels of LDL are associated with its Receptor (LDL-R). The study of ergosterol-rich extracts’ impact on mRNA expression of the proteins involved in lipid metabolism revealed a reduction in FAS and HMG-CoA reductase expression, as well as an upregulation of LDL-R expression after mice on a High-Fat Diet (HFD) were treated with ergosterol [8].

The Sterol Regulatory Element-Binding Proteins (SREBPs) regulate HMG-CoA reductase expression [27]. Animals treated with ergosterol extracts and on HFDs showed a significant increase in fecal excretion of cholesterol and bile acids (BAs), along with the inhibition of SREBP-1c and HMG-CoA reductase expression [28].

Ergosterol and plant phytosterols’ hypocholesterolemic properties are generally supported by the decrease in cholesterol absorption due to the competition mechanisms associated with cholesterol structural similarity. Thus, these mechanisms remain unclear, and the regulation of sterols’ mRNA expression can elucidate their target bioactivity. Ergosterol extracts can up-regulate LDL-R mRNA expression in intestine Caco-2 cells and modulate the expression of other genes related to the lipid metabolism in liver HepG2 cells (e.g., inhibition of HMG-CoA reductase and down-regulation of diglyceride acyltransferase DGAT1) at 6.25 μL/mL of extract after in vitro digestion [25].

### 2.2. Policosanol

Policosanol is a mixture of long-chain saturated fatty alcohols whose main constituent is octacosanol (CH_3_(CH_2_)_26_CH_2_OH). Natural sources comprise rice bran, wheat, sugarcane, and beeswax [4]. For instance, in wheat (*Triticum aestivum* L.), it is described that policosanol content may be up to 812.3 mg/100 g [29]. The commercial mixture of fatty alcohols was first developed by Dalmer Laboratories (Havana, Cuba) in 1991, extracted from sugarcane (*Saccharum officinarum* L.) wax [30].

Policosanol has captured interest since 1993, when it was proven that oral administration leads to octacosanol accumulation in the liver and in the adipose tissues, especially in the brown adipose tissue [31]. It has been proposed that octacosanol metabolization, oxidation, and degradation to FAs can be via β-oxidation, which can stimulate the conversion of lipids into energy [31]. After supplementation of 30 mg/day of octacosanol for 4 weeks in 10 healthy women, results revealed an up-regulated hepatic BA synthesis and excretion. These findings indicate a possible interaction between cholesterol and octacosanol metabolism, since BA derives from cholesterol [32].

In investigations assaying HFD-fed mice to promote high body fat gain, treatment with octacosanol or policosanol showed lower body fat gain, prevention of insulin resistance, and modulation of hepatic lipid content [4]. Authors proposed that octacosanol may impact body weight by reducing the expression of genes involved in hepatic lipogenesis as FAS, as well as in cholesterol uptake. The upregulation of thermogenic and β-oxidation related genes (Peroxisome proliferator-activated receptor: PPARgc1-α, Free Fatty Acid Receptor 4: FFA-R) has been also achieved after octacosanol treatment [4]. Other studies performed on hyperlipidaemic mice models supplemented with policosanol observed reduced serum levels of TGs and increased Peroxisome Proliferator-Activated Receptor-g (PPAR-g) [33].

Furthermore, octacosanol supplementation (40 mg/day for six days) in athletes subjected to caloric restriction and exercise training, revealed an improvement in the lipid profile, associated with an increase in HDL plasma levels and decrease in LDL and TGs, accompanied by a reduction in oxidative stress (increase in superoxide dismutase—SOD) [34].

The hepatoprotective potential of policosanol on NAFLD mice models led to a reduction in hepatic oxidative stress by reducing Malondialdehyde (MDA) and Thiobarbituric Acid Reactive Substances (TBARS) and increasing glutathione (GSH) protein content. Furthermore, policosanol can be compared to known drugs such as silymarin in terms of its hepatoprotective effects [35].

After reviewing the literature, other authors evidenced that policosanol induces AMP-activated protein kinase (AMPK) phosphorylation and inhibits HMG-CoA reductase [36].

Assays on hepatoma (HepG2) cells revealed that policosanol (at 50 μg/mL) increased the phosphorylation of AMPKsphingo and the mRNA downregulation of HMG-CoA reductase [37]. The inhibitory effect of policosanol on cholesterol biosynthesis in other cell lines rather than hepatocytes has been also studied. Hence, when fibroblast cells were exposed to a lipid-free medium, de novo synthesis of HMG-CoA reductase was observed [38].

Randomized placebo-controlled studies carried out on hypercholesterolemia patients after octacosanol administration (approx. 20 mg/day for 6 weeks) revealed approximately 30% significantly lower LDL profiling. Furthermore, animal studies also suggest LDL-enhanced catabolism [30].

### 2.3. Dietary Sphingomyelin

Sphingomyelin (SM) constitutes the most abundant sphingolipid in cell membranes and lipoproteins composition. However, endogenous SM has been associated with atherogenesis development (a disorder in the artery wall characterized by an inflammation process and lipid accumulation leading to plaques formation), and consequent elevated cardiovascular risk, when at high plasma levels [39]. On the other hand, dietary SM has shown potential to hinder obesity-related metabolic disorders, specially towards cholesterol homeostasis. This section aims to review the main beneficial effects of dietary SM on obesity disorders, its mode of action, and its suitability for humans.

Several animal model studies have demonstrated the benefits of dietary SM against dyslipidaemia. In fact, male C57BL/6 mice fed a HFD supplemented with chicken egg or bovine milk SM have revealed lower weight gain and body weight, reduced serum cholesterol and non-esterified FA levels and decreased liver cholesterol and TGs amounts, compared to mice fed only HFD [12,13,40]. Dietary SM has also been associated with anti-atherogenic properties. The genetically modified apolipoprotein E-knockout (ApoE^−/−^) mice exhibit poor lipoprotein clearance, being prone to atherosclerosis development [41]. When male ApoE^−/−^ mice were fed HFD and 0.1% (*w*/*w*) egg SM for 8 weeks, their aortic root lesion area was reduced compared to mice without SM supplementation [42]. Moreover, female ApoE^−/−^ mice, which are more susceptible to atherosclerotic progression than males [43], revealed a reduction in atherosclerotic lesion area after a 19-week treatment with normal chow diet and 1.2% (*w*/*w*) egg SM, compared to mice not receiving dietary SM [44]. Beside dyslipidaemia and atherogenesis, dietary SM has also demonstrated to counteract adipose tissue inflammation, another obesity-related metabolic complication [45]. After feeding male C57BL/6 mice with a HFD and 0.1% (*w*/*w*) egg SM for 10 weeks, Norris et al. [12] observed a decrease in serum CCL2 (chemokine factor related to macrophages’ recruitment into adipose tissue), and in epididymal adipose tissue macrophages’ infiltration, along with a reduction in the mRNA expression of inflammatory markers—F4/80, Cd68, Cd11c, Ccl2 and TNF-α—compared to the control (with no Sphingomyelin supplementation).

Research works carried out with animal models were usually performed with SM from chicken egg or bovine milk, which contain 1.90–2.54 mg/g and 11.7–130 mg/L, respectively [46]. These differ in sphingosine base and amide-linked FA. Egg SM species exhibit a more uniform composition, with principally palmitic acid (C16:0) and d18:1 base [47]. On the other hand, milk SM composition is more variable, not only in terms of FA (C16:0, C22:0–C24:0) but also in terms of sphingosine base (d16:0–d19:0 and d16:1–d19:1) [48]. Norris et al. [40], who fed male C57BL/6 mice with a HFD and 0.25% (*w*/*w*) SM for 4 weeks, observed that, contrary to milk SM, egg SM supplementation led to an increase in serum cholesterol and liver TG levels, beside also increasing epididymal fat weight and serum TGs, compared to the control. In contrast, a further study by this same research team [12], where the same mice model received a HFD plus 0.1% (*w*/*w*) of egg or milk SM for 10 weeks, showed that egg SM was generally more effective than milk SM in reducing cholesterol and fat levels. Authors claimed that differences between egg and milk SM effectiveness might be related to amide-linked FA chain length and sphingosine base saturation, but also stated that contrary effects could be associated with cholesterol/SM ratio in animal’s diet, as in the last above-mentioned study [12] was added 0.15% (w/w) cholesterol to mice HFD, contrary to the previous one [47]. Cholesterol and SM strongly interact with each other [49], and Chung et al. [13], who supplemented mice HFDs with cholesterol (0.15%, *w*/*w*), reported beneficial effects towards liver lipid levels by egg SM as well. In the meantime, milk SM has been demonstrated to be effective even with no cholesterol supplementation [40,50]. Taken altogether, it seems that dietary SM effectiveness against dyslipidaemia is influenced by the cholesterol ratio in the diet, depending on SM compositional structure.

Some studies have reported increased levels of faecal cholesterol after SM supplementation to mice, which suggested that dietary SM would counteract cholesterol body levels by reducing its intestinal absorption [13,50]. In fact, a research work carried out on intestinal epithelial Caco-2 cells observed that egg SM treatment (0.6 mmol/L), and added cholesterol (0.1 mmol/L), for 21 days, led to a lower cholesterol transport rate than the control, driven by the reduction in micellar cholesterol incorporation and cholesterol micellar solubility [51]. The authors observed that SM might also act through the down-regulation of the epithelial cholesterol uptake transporter NPC1L1 expression, suggesting that it could be related to the decrease in SREBP-1 and SREBP-2 gene expression. When acting on the liver, dietary SM may lower hepatic cholesterol levels by further inhibiting circulating cholesterol absorption through a reduced expression of the liver X receptor α (LXRα) and down-stream target genes, such as ATP-binding cassette ABCA1, ABCG1, ABCGA5, and ABCG8 proteins (involved in cholesterol efflux), and LDL-R (involved in cholesterol uptake) [13]. Moreover, the reduction in hepatic TGs by dietary SM has been associated to the suppression of the LXR-SREBP-1c regulated FA synthesis pathway [13], and to the reduced expression of the PPARg-related genes involved in TG synthesis, like stearoyl-CoA desaturase 1 (SCD-1) [12]. The SCD-1 is involved in de novo lipogenesis, and the inhibition of its activity can reduce susceptibility to obesity [52].

There are studies reporting health benefits after dietary SM treatment in animal models that applied doses equivalent to a daily consumption of approximately 405 mg to 1.5 g of SM to 70 kg-person [12,40]. Ohlsson et al. [53] tested a milk drink enriched in polar lipids from buttermilk on healthy adult male and female subjects, providing 38 mg cholesterol and 700 mg SM per day for 4 weeks. An analysis of the plasma lipid levels revealed no significant differences from the control (given a placebo drink). The only considerable alteration observed was on plasma apolipoprotein A1 (ApoA1) level, which was lower than baseline. The ApoA1 is the predominant constituent of HDLs, contributing to the reverse cholesterol transport pathway (from peripheral cells to the liver for further excretion) [54]. A further study carried out by the same above-mentioned research team on healthy male subjects [55], to whom the same milk drink was given, along with a standard breakfast containing an additional 130 mg cholesterol, reported no changes on post-prandial plasma lipid levels compared to the control. Ramprasath et al. [56] claimed that one major weakness of these last studies was not being well controlled with a background diet. Therefore, in this former research work a controlled diet was provided (240 mg cholesterol/day) supplemented with milk SM (1 g/day) to healthy adult male and female subjects for 2 weeks. Nevertheless, the only significant difference observed was the increased serum HDL cholesterol, compared to subjects not receiving SM. One factor that could have affected dietary SM efficiency is that SM hydrolysis in humans is faster and more efficient than in rodents, as SMase, the enzyme responsible for SM digestion, is expressed not only in the intestine but also in the liver (into the bile) of humans [57]. On the other hand, among the different limitations in the above-mentioned studies, the fact that the trials were carried out with healthy subjects stands out. Indeed, when wild-type male mice, with no HFD-induced obesity, were treated with milk SM (1%, w/w of diet), no considerable differences were observed in lipid levels [50]. Thus, it seems that dietary SM has the potential to treat dyslipidaemia only in subjects with metabolic disorders. This being said, further studies on human subjects with dyslipidaemia conditions (e.g., overweight or obese subjects) should be carried out in the future.

In conclusion, dietary SM shows the potential to hinder obesity-related metabolic disorders, especially dyslipidaemia, the effectiveness of which is affected by SM compositional structure and cholesterol ratio. The SM promotes cholesterol homeostasis by reducing its absorption in the intestine and liver. Nevertheless, dietary SM seems to be effective only towards a metabolic disorder state, given the lack of beneficial effects in healthy subjects.

## 3. Prevalence and Current Impact of Mental and Neurological Disorders

Neurological and mental disorders are prevalent worldwide and are a leading cause of disability and death [58,59,60]. According to the Global Burden of Disease study, in 2019, there were nearly 10 million deaths and 349 million DALYs (disability-adjusted life years) due to neurological disorders globally [59]. In Europe, according to a report by the European Academy of Neurology, more than half of the population suffers from a neurological disease [61]. The worldwide burden of neurological disorders accounted for 280 million DALYs in 2017, and neurological disorders ranked third for DALYs in the EU28 [58]. In 2016, neurological disorders were responsible for 276 million DALYs and 9 million deaths globally [60]. Mental disorders are also prevalent worldwide, with around a quarter of the population in many European countries reporting suffering from at least one mental health condition [62]. Anxiety disorders are the most common mental disorders across EU countries, affecting an estimated 25 million people or 5.4% of the population [59]. Mortality rates associated with mental and neurological disorders are higher than those of the general population [63]. The prevalence, incidence, and death rates of neurological and mental disorders can vary by country and region, and data can be found online on the database of the Institute for Health Metrics and Evaluation (IHME), in the Global Burden of Disease results tool (https://ghdx.healthdata.org/gbd-results-tool, accessed on 18 January 2023).

Neurological disorders include stroke, neonatal encephalopathy due to birth asphyxia and trauma, migraine, Alzheimer’s disease (AD) and other dementias, meningitis, idiopathic epilepsy, brain and central nervous system (CNS) cancer, neural tube defects, head injuries, Parkinson’s disease (PD), spinal injuries, encephalitis, tension-type headache, other neurological disorders, tetanus, Down syndrome, multiple sclerosis, and motor neuron disease [59].

Neurodegenerative diseases (NDs) are a group of chronic and progressive disorders that affect the CNS and result in the degeneration and eventual death of neurons [64]. Examples of NDs include AD, PD, and Huntington’s disease (HD). Despite extensive research, there are currently no effective treatments for NDs, and therapy options only alleviate symptoms.

Overall, the incidence of NDs is increasing due to aging populations in many countries. While there is currently no cure for most NDs, ongoing research is working towards better treatments and prevention strategies. Recent studies have suggested that alpha-Tocopherol, phospholipids, butyrate and its derivative, sodium butyrate, may have therapeutic potential for mental and neurological disorders.

### 3.1. α-Tocopherol

Alpha-Tocopherol (α-Toc) is a plant-derived fat-soluble vitamin, consisting of a chromanol ring bonded to an aliphatic side chain [65], and is the most bioavailable form of vitamin E (Vit E) [66]. Vit E, described for the first time by Evans and Bishop in 1922 [67], is the term used to designate the set of four tocopherols and four tocotrienols (α, β, γ, and δ), eight fat-soluble vitamers [68]. Among these, α-Toc is the most abundant isoform in plasma, despite not always being the major form in foods; in some foods, γ-Tocopherol is present in higher levels than α-Toc [69]. Because animals lack the enzymatic system required to synthesize α-Toc, it can be obtained only via diet. The Food and Drug Administration (FDA) recommends that the daily intake of Vit E should be 15 mg for adults and children over 4 years old [70]. On the other hand, the European Food Safety Authority (EFSA) [71] established an adequate intake of 13 mg/day for adults and children over 10 years old, 5 mg/day for children from 7 to 11 months, 6 mg/day for children from 1 to 3 years, and 9 mg/day for children from 3 to 10 years old. Novotny et al. [72] reported that the estimated average requirement is defined as the intake capable of mitigating hydrogen peroxide-induced hemolysis, being 12 µmol/L of α-Toc in tissues. To meet this content in the tissues, a 12 mg/day of α-Toc should be provided by diet. High amounts of α-Toc can be found in raw hazelnuts, almonds, and safflower and sunflower oils [65,67,70]; while, in other oils, like apricot oil, and seed oils, as hemp seed oil, or nuts like peanuts and walnuts, higher amounts of γ-Tocopherol are found (Table S1) [65,70]. In fact, γ-Tocopherol is highly present in the western diet; on the other hand, regarding synthetic food supplements, α-Toc is usually the main isoform of Vit E [65].

Alpha-Tocopherol is absorbed by the intestine to be stored in the liver, similarly to other fat-soluble vitamins [66,67,69]. Since all the Vit E isoforms are stored in the liver, why is α-Toc the most prevalent in plasma if it is not always the prevalent Vit E isoform in dietary intake? This fact is attributable to the α-Tocopherol transfer protein (α-TTP) that preferentially binds to α-Toc isoform [69]. Alpha-TTP shows different affinities depending on the Vit E isoform: 100% for RRR-α-Tocopherol, 30% for β-Tocopherol, 12% for α-tocotrienol, 11% for SRR-α-Tocopherol, 9% for γ-Tocopherol, and 2% for δ-Tocopherol. Alpha-Tocopherol is the most prevalent Vit E isoform in plasma, probably due to the affinity of α-TTP for α-Toc, that returns α-Toc to the plasma [67]. Studies showed that, when α- and γ-Tocopherol are equally fed together, initially, their content in plasma is the same; however, after 24 h, α-Toc is more prevalent than γ-Tocopherol, due to the affinity of α-TTP to α-Toc [69]. All Vit E forms are stored in the liver and have several possible destinations: a) α-Toc can be transported to and from the periphery by means of α-TTP and lipoproteins; b) it can be excreted in the bile; c) it can be transformed into 2,5,7,8-tetramethyl(3′ carboxyethyl)-6-hydroxychromanol (α-CEHC) to be excreted in the bile or to be secreted in the plasma towards the kidneys to be excreted through urine; d) it can be oxidized by a peroxyl radical; or e) it can remain in the liver in lipid droplets [73]. Alpha-Tocopherol is the only Vit E form that is selectively transported to plasma by lipoproteins such as very low-density lipoprotein (VLDL), LDL, and HDL, facilitated by the action of α-TTP [69].

Alpha-Tocopherol is absorbed from the intestine and transported through the lymph to the liver via chylomicrons. In the liver, α-Toc will be stored in the endosomes, where it will be captured later by α-TTP, forming α-TTP-α-Toc complex. This complex will release α-Toc in the liver membrane to be excreted to plasma lipoprotein by means of newly formed VLDL, LDL or HDL [67] spontaneously or through utilizing the machinery for intracellular cholesterol transport protein ABCA1. There is evidence that ABCA1 is a major exporter of α-Toc towards the outside of the cell [69]. Alpha-TTP returns α-Toc to the plasma, protecting it from the lysosomal degradation pathway and, ultimately, forming the biliary excretion [67,73]. This transport protein is highly expressed in the liver and, in lower levels, in other tissues like rat brain, spleen, lung, kidneys, and retina [69]. In fact, α-TTP was found in five different brain regions: cortex, cerebellum, hippocampus, brainstem, and midbrain [74]. In the brain, α-Toc will be transported to neurons by α-TTP action [75] or will be selectively delivered to CNS in HDL particles via scavenger receptor class B type 1 (SRB1) present in the blood–brain barrier (BBB). Using liver-like mechanisms, α-Toc is transported from the BBB to astrocytes. There, Apolipoprotein E (ApoE), synthetized by the astrocyte, will transport α-Toc between various CNS cell types through the central spinal fluid, similar to the cholesterol movement [76]. Mutations in α-TTP expressed in the liver can cause the dysregulation of plasma α-Toc, resulting in low levels of plasmatic α-Toc. Patients who exhibit low levels of plasma α-Toc, even in the presence of normal levels of other lipids, suffer from Ataxia with Vitamin E Deficiency (AVED) [69]. This is a rare autosomal-recessive disease due to a mutation in the α-TTP gene that causes progressive cerebellar ataxia, dysarthria, and progressive clumsiness [77], being directly linked to major depressive disease [78]. Huang et al. [79] demonstrated that α-Toc can enhance the autophagy in the prefrontal cortex, showing antidepressant effects by feeding female mice with 50 and 75 mg/kg of α-Toc for 2 weeks. These antidepressant effects, due to the presence of α-Toc, were also confirmed by Manosso et al. [80]. In the latter study, the acute administration of α-Toc (3 doses of 30 and 100 mg/kg) to female mice combined with the administration of TNF-α (0.001 fg/site, i.c.v), showed that TNF-α stimulated depressant effects that could be prevented by the pre-administration of α-Toc. In the same study, the combination of acute administration of α-Toc with antidepressant compounds, like fluoxetine, imipramine, and bupropion, showed a synergic effect. Even in the presence of an inflammatory condition, such as cancer or chronic hepatitis C, α-Toc improved the effectiveness of the conventional antidepressant compounds.

The antidepressant effect was verified not only in the acute administration (α-Toc 10, 30, 100 and 300 mg/kg 60 min before testing) but also in a long-term administration (α-Toc 10–100 mg/kg, for 28 days). Long-term administration enhanced the antioxidant effect in the prefrontal cortex and hippocampus, contributing to the prevention of oxidative damages in the brain, which is associated with the progression of major depression disease [81]. These findings were not only observed in pre-clinical studies but also in clinical studies; by monitoring the dietary intake and the evaluation of the depression symptoms of several subjects, it was possible to establish a relationship between these two factors. In these studies, a dietary assessment, a register of all food and beverages consumed, was carried out by answering a phone questionnaire every 24 h. The obtained data were converted in terms of macro and micronutrients by software conversion. The assessment of depression symptoms was also performed by survey and classified according to the Geriatric Depression Scale (GDS). The biochemical measurements, the quantification of other lipids than α-Toc in plasma samples, was also carried out for total cholesterol, TGs, HDL, and LDL [82,83]. These biochemical measurements do not control depression markers, such as cortisol and serotonin [84]. The conclusions of these studies showed that high intake, or high levels in blood samples, of antioxidants (i.e., α-Toc) have been reported to have favorable effects on cognitive and neural functions [82,83]. This may be due to the capacity of antioxidants to protect the synaptic membranes from oxidation, preserving plasticity [82]. On the other hand, a poor daily diet can result in low levels of α-Toc, being related with the deterioration of functional health and chronic diseases [78]. This implies reduced antioxidant protection against lipid peroxidation, leading to an increased risk of major depression [85]. By following the dietary patterns of 794 men (81.4 years old on average) for 5.5 years, it was possible to verify that, despite the normal values of TGs and LDL cholesterol, these showed significant depletion of α-Toc. At the same time, an association between poor antioxidant intake and increased depressive symptoms was also established [86]. The administration of α-Toc doses (400 IU once, twice, and thrice a day for 4 weeks to 15 patients) showed a decrease in symptoms such as anxiety, apathy, and depression, being associated with a free radical mechanism [87]. A co-supplementation of α-Toc (400 IU/day) with omega-3 FAs (1000 mg/day) for 12 weeks showed successful results in enhancing mental health parameters in 40 women with increased risk of depression [88]. Some studies have proven that there may be a possibility of using α-Toc as an adjuvant in the treatment of depression; however, more studies will be needed to determine the safety of this approach [89].

In summary, α-Toc is a plant-derived isoform of Vit E, that can be consumed by the intake of sunflower oil, hazelnuts, and almonds. In order to attain the adequate intake of 13 mg/day recommended by EFSA [71], it would be necessary to ingest about 26 g of sunflower oil, 50 g of hazelnuts or 45 g of almonds per day [70]. Alpha-Toc can be stored in the liver, and will be transported to plasma, and brain tissues, mediated by α-TTP, which has a preferential affinity for this isoform. A relationship between α-Toc level and cognitive function or brain health has been established [76]. Alpha-Toc has been shown to be able to reduce and prevent depressive symptoms, mainly as a result of its antioxidant properties. This effect was verified even when the uptake was in an acute or long-term administration. At the same time, a synergic effect was observed by improving the effectiveness of some classical antidepressant compounds.

### 3.2. Cholesterol and Phospholipids from Krill Oil

This section aims to review the role of cholesterol in human biochemistry, mainly at the neurological level. From a nutritional point of view, during the last 50 years, it has been recommended that this lipid be strictly controlled to ensure good health and longevity. However, recent research shows that cholesterol is essential for proper brain functioning, particularly during senescence. Finally, the existing scientific evidence on using lipids to restore cholesterol homeostasis in the brain will be discussed.

In 1938, Carl Müller observed that among deceased patients suffering xanthomatosis (lipid accumulation under the skin surface), the cause of death was circulatory system disorders and he hypothesized that circulating lipids might accumulate not only in the skin but in other body structures, such as arteries [90]. Later, Ancel Keys, using data from the seven country study, found an association between serum cholesterol (in addition to age, blood pressure, and smoking) and cardiovascular death risk [90]. Since then, blood cholesterol concentration has become one of the markers of cardiovascular health and disease risk in general, it being recommended that total levels in the blood should not exceed 200 mg/dL. At the same time, lipoprotein values should be less than 100 mg/dL for LDL and above 40 mg/dL for HDL. Blood TGs should not exceed 150 mg/dL.

However, as pointed out elsewhere, several clinical trials suggest a paradox between mortality, cholesterol, and aging [91]. Thus, meta-analysis studies with individuals aged 62 years reported a linear relationship between LDL, cardiovascular events, and mortality; on the other hand, a systematic review of cohort studies concluded an inverse relationship between LDL and mortality, which seems to be confirmed by follow-up cohort trials on 83- and 78-year-old individuals, where no significant association was found between baseline cholesterol levels (HDL, LDL, total, and TGs) and mortality. Interestingly, in the latter study, the authors found that decreased cholesterol synthesis and absorption in the older group correlated with higher mortality risk [91]. These results suggest biochemical or epigenetic changes after the age of 70.

One of the main effects of aging is the worsening of cognitive functions, which, in the case of dementia, leads to the loss of memory and abstract thinking directly related to alterations in the CNS.

With a weight equivalent to 2% of the body, the human brain contains 20% of total cholesterol [92] and 70% of this amount is found in myelin sheaths [93]. This material, produced and maintained by oligodendrocytes in the CNS and Schwann cells in the peripheral (PNS), is composed primarily of lipids (i.e., 28% phospholipids [PLs], 21% of both cholesterol and glycolipids) and is essential for nerve impulse transmission [94]. In this sense, myelin wraps around the neuron’s axon (multilamellar structure) for a given length and acts as an insulator, while the exposed regions (i.e., node of Ranvier) are repeaters of the electrical action potential [94]. In addition to nerve signal forwarding, cholesterol is also involved in synaptic transmission. When this impulse reaches the synapse (i.e., neuronal junction), it opens voltage-gated Ca^2+^ channels causing vesicles containing neurotransmitters to fuse with the membrane, releasing them into the synaptic cleft [95]. In these processes, cholesterol is involved in both the exocytosis and release of neurotransmitters (pre-synaptic events) and the presence of receptors (post-synaptic events).

Thus, the disruption of the homeostatic balance of cholesterol will result, or will be involved, in brain diseases that affect the normal functioning of neurons.

Any structure is susceptible to degradation, and in the case of myelin, this leads to the release of residues that are removed by both microglia (cells of the nervous system responsible for protecting it) and bone-marrow-derived macrophages (BMDM) via the endosome-lysosome pathway [94]. But, over the course of an individual’s life, changes occur, probably epigenetic [96], which result in the cells responsible for the removal of myelin debris becoming less efficient and their components (mainly cholesterol) accumulating inside the cell [93,97].

This, in turn, causes precipitation of cholesterol in the form of crystals (which can rupture the lysosome and trigger apoptosis processes), activation in these cells of pro-inflammatory states where homeostasis-related genes are inhibited (e.g., disease-associated microglia; DAM), and interference with remyelination processes so that they are performed poorly [93,94].

The mechanisms that maintain cholesterol homeostasis in the brain are based on eliminating its excess. The BBB allows the strict control of substances entering the brain, and, as a result, all cholesterol in the brain comes from de novo synthesis [95]. In contrast, the elimination of the excess of cholesterol from the brain takes place in the liver, where it arrives as oxysterols (i.e., 24(S)-hydroxycholesterol; 6–12 mg/d) transported by ApoE [95].

In the human genome, there are three alleles for this lipoprotein gene (i.e., ε2, ε3, ε4), with homozygous (i.e., ε3/ε4) and heterozygous (i.e., ε3/ε4, ε2/ε4) individuals having a 30–35% and 20–25% risk, respectively, of developing AD [98]; it has been described that ApoE4 (i) has a low affinity for HDL, resulting in neurotoxic ApoE4 aggregates. Of the three forms, it binds poorly to 24(S)-hydroxycholesterol and this, ultimately, results in the accumulation of cholesterol, mainly in oligodendrocytes, leading to impaired myelination [98,99]. However, it has been recently shown that restoring cholesterol clearance mechanisms helps to recover ApoE4-disrupted homeostasis and improves cognitive processes in mice [99].

Since the data obtained to date suggest that lipids are related to dementia and cognitive processes, the role of ApoE in the development of AD has been approached from the possible differences in lipid composition associated with the various alleles of this lipoprotein.

In the CNS, ApoE is produced in the endoplasmic reticulum of astrocytes, which are the major producers, and subsequently glycosylated and sialylated in the Golgi organelle [100]. In order to perform its function, ApoE has to be lipidated (i.e., addition of a lipid group to the peptide chain) by the membrane transporter proteins ABCA1 and ABCG1 [100]; this results in the attachment of cholesterol and PLs to nascent ApoE to give lipoprotein particles with HDL-like size and density [101,102]. In dyshomeostasis, where lipid accumulation can occur in the form of droplets in stressed astrocytes (i.e., as in AD [103]), the result will be TG-rich lipoproteins [102]. In addition to cholesterol, PLs and TGs, cholesterol esters (CE) can also be found in ApoE [104], but studies comparing the lipid profile of ApoE3 vs. ApoE4 showed that the PLs glycerophosphatidylcholine (PC), glycerophosphatidylinositol (PI), glycerophosphatidylserine (PS), and glycerophosphatidylethanolamine (PE) were significantly reduced in the dementia-associated apolipoprotein [105].

The focus in the search for therapeutic targets has recently turned to the membrane receptor expressed on microglia (i.e., CNS) and macrophages (i.e., periphery) called triggering receptor expressed on myeloid cells 2 (TREM2). It has been described as being associated with removing myelin debris through the transcription of genes related to lipid catabolism, phagocytosis, and the regulation of ApoE expression [106]. These same studies have also observed that pathogenic variants of TREM2 show defective binding to PLs, pointing, again, towards the role of these lipids in normal brain function.

From the discussed studies, it can be inferred that, for the good functioning of cognitive abilities, it is crucial to maintain healthy cholesterol levels. Thus, in the case of high concentrations, the strategy is to act on targets that re-establish brain cholesterol homeostasis, and finally, PLs emerge as compounds capable of acting on these targets (i.e., ApoE, TREM2). Taken together, this could be summed up by the idea that what is good for the heart is also good for the head. If omega-3 FAs are effective in maintaining cardiovascular health, it seems logical to think that, in this context, they may be useful in maintaining cognitive well-being.

It is interesting to note that docosahexaenoic acid (DHA) accounts for 30% of the lipids in the brain, and studies in Alzheimer’s patients ingesting doses of 430 mg DHA/d and 150 mg/d of eicosapentaenoic acids (EPA) for 6 weeks resulted in a decrease in phosphorylated tau protein [107] as well as an improvement in the Mini-Mental State Test [108]. These results probably relate to the fraction of free FAs in the oil tested. While TGs (i.e., the predominant lipid in oils) are hydrolyzed and subsequently re-esterified in the digestion process and carried by chylomicrons to the liver, PLs and FAs (the latter by association with albumin) can be transported directly into the bloodstream [109] and are able to diffuse through the BBB, in the case of FAs, [110] or via a membrane transporter (i.e., Major facilitator superfamily domain-containing protein 2—MFSD2A) in the case of PLs [111].

Recently, krill oil has been the subject of several investigations due to its high PL concentration (>45%) where, of the total amount, it contains 25% of EPA and 4% of DHA [112]. Indeed, administering Krill oil (100–500 mg/kg/d) to mice injected with beta-amyloid protein (Aβ) resulted in improved learning and memory performance, which appeared to be related to a decrease in neuronal oxidative stress and apoptosis [113]. These findings are confirmed by results suggesting an improvement in synaptic activity after feeding mice expressing human ApoE4 with Krill lysophosphatidylcholine (LPC; 1.2 µg/g diet) [114]. In parallel, a dose of 2 g/day in 67-year-old volunteers showed an improvement in memory functions by activation of the dorsolateral prefrontal cortex, which the authors interpreted as a preventive protective effect on cognitive function [115].

Phospholipids rich in long-chain omega-3 from Krill oil may be a dietary supplement that can help, combined with healthy lifestyles, to prevent the cognitive decline associated with aging. However, there are no available data on the impact on brain cholesterol and its homeostasis which, if confirmed, would provide solid evidence of its usefulness.

### 3.3. Butyrate and Sodium Butyrate

Recent studies have suggested that butyrate and its derivative, sodium butyrate (NaB), may have therapeutic potential for NDs.

Butyrate is a short-chain fatty acid (SCFA) that is produced by gut bacteria as a result of dietary fiber fermentation in the colon [116]. It is an important energy source for colonocytes and has been shown to have anti-inflammatory and anti-tumour effects [117,118]. Butyrate also plays a crucial role in maintaining gut homeostasis and the gut-brain axis, which has been implicated in the pathogenesis of NDs.

Almost all of the butyrate generated is metabolized in the colonocyte (90%) and the part not metabolized is carried into the liver and used as an energy source for hepatocytes. However, SCFA can reach the brain and cross the BBB [119]. The BBB is a physical barrier that separates circulating blood from the brain extracellular fluid. It is composed of brain endothelial cells and sealed-tight junctions including claudins and injuryng [120].

Sodium butyrate is the sodium salt form of butyrate and studies carried out by Sun et al. [121] have demonstrated that NaB has neuroprotective effects in mice with cerebral ischaemia/reperfusion injury. In an attempt to understand the molecular mechanisms involved in the protection of the BBB rupture, Li et al. [120] showed that mice (C57BL/6) with induced brain injury, when treated with NaB, revealed attenuated neurological deficits, brain oedema, neuronal degeneration, and BBB damage, as well as an increased expression of tight junction-associated proteins in the brain [120].

Other authors [122] demonstrated that NaB can ameliorate PD symptoms in C57BL/6J mice by the activation of GPR109A (a G protein-coupled receptor), induced by lowered activity of the NF-kB signalling pathway. The activation of GPR109A can reverse the damage on the BBB which is involved in the pathogenesis of PD.

One of the hallmarks of NDs is neuroinflammation, which is characterized by the activation of glial cells (microglia and astrocytes) and the production of pro-inflammatory cytokines. Studies have shown that butyrate and NaB can modulate neuroinflammation by inhibiting the activation of glial cells and reducing the production of pro-inflammatory cytokines, such as TNF-α and interleukins 6 (IL-6) and 1β (IL-1β) [116]. These authors also demonstrate that NaB can attenuate pro-inflammatory cytokine expression in microglia in aged mice.

Butyrate has also been shown to increase the expression of anti-inflammatory cytokines such as interleukin10 (IL-10). These anti-inflammatory effects of butyrate, that may also be explained by the fact that it is a histone deacetylase (HDAC) inhibitor, may help to reduce neuronal damage and slow the progression of NDs [116,123]. Similarly, NaB also inhibited the activation of microglia and the occurrence of neuroinflammation and reduced the accumulation of Aβ in an animal model of AD using 5XFAD mice [124].

Another hallmark of NDs is mitochondrial dysfunction, which can lead to oxidative stress and neuronal cell death. Mitochondria are major intracellular sources of reactive oxygen species (ROS) [125], that play an important role in the activation of nuclear factor NF-kB, which triggers inflammation, leading to oxidative stress [126]. Studies performed by Rose et al. [127] have shown, for the first time, that butyrate can improve mitochondrial function by increasing the expression of mitochondrial biogenesis genes and reducing oxidative stress in lymphoblastoid cell lines (LCLs) from children with autism. Sodium butyrate has also been shown to have a protective effect against oxidative stress. Xing et al. [128] found that NaB protected against oxidative stress in liver HepG2 cells by modulating the nuclear factor erythroid 2-related factor 2 (Nrf2) pathway and mitochondrial function; Nrf2 is a transcription factor that regulates the antioxidant response.

Furthermore, NaB has also been shown to improve motor function in animal models of PD by protecting dopaminergic neurons from degeneration via the stimulation of glucagon like peptide-1 (GLP-1) secretion [129]. Moreover, NaB was shown to improve cognitive function in mice with induced AD by regulating the metabolism of astrocytes [130].

In addition to its effects on neuroinflammation and mitochondrial function, butyrate and NaB have also been shown to have epigenetic effects that may be relevant to the pathogenesis of NDs. Epigenetic modifications, such as DNA methylation and histone acetylation, play a crucial role in regulating gene expression and have been implicated in the pathogenesis of NDs. Studies have shown that butyrate and NaB can modify histone acetylation and gene expression in neuronal cells [131,132]. These epigenetic effects may help to restore the expression of genes that are important for neuronal function and survival and may help to slow the progression of NDs.

Short-chain FAs activate cells through G-protein-coupled receptors (GPRs) such as GPR41 and GPR43, which have been renamed as free fatty acid receptor FFRA3 and FFRA2, respectively. Another butyrate binder is GPR109A [133,134].

Sodium butyrate was shown to activate, in neurons, the GPR41-Gβγ-P13K/Akt pathway and attenuate apoptosis, improving neurological function in a rat model [135].

An additional receptor that can be used as a therapeutic target for NDs is the gamma-aminobutyric acid (GABA_A_) receptor—a type of neurotransmitter receptor found in the CNS. It plays a critical role in regulating neuronal activity and is the target of many drugs used to treat anxiety, insomnia, epilepsy, and other CNS disorders [136].

In conclusion, butyrate and NaB have been shown to have therapeutic potential for NDs. These compounds have anti-inflammatory, antioxidant, and epigenetic effects that may help to reduce neuronal damage and slow the progression of ND diseases. While more research is needed to fully understand the mechanisms underlying the therapeutic effects of butyrate and NaB on the brain, these compounds hold promise as potential therapeutic agents for NDs. However, it should be noted that the use of these compounds for the treatment of NDs is still in study, and more research is needed to establish their safety and efficacy in humans.

## 4. Empirical Rules for Bioactivity Determination and In Silico QSAR

This section aims to briefly introduce the reader to the main parameters and descriptors that can help in the selection of molecules with potential bioactivity for the further study and development of drugs, functional foods, or nutraceuticals. The drug discovery and development process is a complex, time-consuming, and costly endeavour: it requires testing hundreds of molecules to obtain reliable models or to find promising candidates. To successfully bring a new therapeutic compound to the market, researchers must navigate multiple stages, including target identification, lead optimization, preclinical evaluation, and clinical trials. In the early stages of drug discovery, it is crucial to identify and prioritize compounds with promising bioactivity profiles to streamline the development process.

In this sense, the application of in silico QSAR models is extremely relevant since, quickly and economically, it allows a first screening of the most promising compounds.

Some QSAR models are more focused on the physico-chemical properties of compounds. This is particularly useful when online databases (i.e., PubChem [137,138], Chemspider [139], OCHEM [140,141], NPASS [142]) do not provide such data. Their utilization is user-friendly since the search can be carried out using the name or Simplified Molecular-Input Line-Entry System (SMILES) of the compound. Such software are equipped with powerful databases that allow them to quickly provide an enormous amount of information related to the physico-chemical properties of the compound under study.

Primarily, regarding the physico-chemical properties of the compounds, there are some empirical rules, such as Lipinski’s Rule of Five [143] or Veber’s rules [144], that assist as valuable guidelines in predicting the pharmacokinetics of the compounds as oral drugs. The most common drugability methodologies, such as the Lipinski, Ghose, Veber, Egan, Muegge, and Leadlikeness parameters, are shown in Table 1. Lipinski’s Rule of Five, established in 1997, is based on the observation that most orally active drugs share certain physicochemical properties that enable them to cross biological membranes and dissolve in both aqueous and lipid environments. In 2002, Veber et al. [144] complemented Lipinski’s guidelines by considering additional properties, such as molecular rigidity and Topological Surface Area (TPSA; meaning the polar surface area of a molecule based on its two-dimensional structure).

Thus, molecules with lower molecular weight (MW < 500 g/mol) are more likely to be absorbed and distributed across biological membranes [145]. Larger molecules may face difficulty in crossing cell membranes, which could negatively impact their bioavailability. Moreover, the partition coefficient octanol-water, logP, represents a measure of a compound’s lipophilicity. As observed by Lipinski et al., values of logP (≤ 5) are more likely to have a balance between hydrophilic and lipophilic properties, enabling them to dissolve in both aqueous and lipid environments and therefore reaching the target site as well as giving an idea of their capacity to penetrate cell membranes [143]. However, further observations have reported that leading compounds in drug discovery have a lipophilicity range of −1 < logP < 3 [146].

Nevertheless, logP per se does not consider the effect of pH (e.g., physiological pH, 7.4) since some molecules can be ionized depending on pH. Thus, logD represents the logarithm of the ratio of the concentrations of all forms (ionized and neutral) of a compound in the organic phase to their concentrations in the aqueous phase; for non-ionizable compounds, logP would be equal to logD. Thus, studies on the oral absorption of different drugs concluded that logD was better related than logP to the logarithmic skin permeability coefficient (logKp) [147,148,149,150]. In ligand efficiency (LE) descriptors, logP can be substituted for logD [146].

Moreover, TPSA is the sum of the surface areas of polar atoms (usually oxygen, nitrogen, and their attached hydrogen atoms) in a molecule, considering their topological environment [144,149]. It dictates the molecule’s interaction with the membrane and Lipinski et al. suggested that its value needs to be within 20 ≤ TPSA ≤ 140 Å^2^. Biological membranes are largely composed of PLs, which have a hydrophobic (nonpolar) tail and a hydrophilic (polar) head. Thus, they are arranged as a bilayer (i.e., head-tail-tail-head) and, for a molecule to diffuse through a membrane, it must interact with both the hydrophobic and hydrophilic regions of the bilayer. Therefore, for optimal membrane permeability, a molecule needs to have a balance between hydrophobic and hydrophilic properties. If a molecule is too hydrophobic, it may have difficulty interacting with the hydrophilic domain of the membrane. At the same time, if it is too hydrophilic, it may have difficulty dissolving in the hydrophobic interior. Studies to estimate the ideal permeability of the BBB found that TPSA values below 90 Å^2^ increased potential penetration [151,152]. This indicates that different organs, tissues, or cells, depending on their composition, may be more or less available for the bioactive compound diffusion depending on its TPSA. Other factors are associated with the flexibility of the molecule. Rotatable bonds are single, non-ring bonds between two non-terminal heavy (non-hydrogen) atoms. Molecules with fewer rotatable bonds tend to have a more rigid conformation, which can negatively affect the molecule-membrane or molecule-protein fitting [153].

Other authors have proposed different metrics to evaluate the effectiveness of a compound as a potential drug candidate [146,154], considering the binding efficiency of a ligand (compound) relative to its size (i.e., LE, ΔG^o^/HA) as well as the correction by lipophilicity (lipophilicity corrected ligand efficiency, LELP, logP/LE) or the relationship of a potency measure (i.e., the necessary amount of drug to produce an effect of a given magnitude) and lipophilicity (i.e., ligand lipophilicity efficiency, LLE, pIC50—logP) [146,147].

These empirical rules work since they capture some of the key molecular features that govern the interaction between a compound and its biological target, as well as its ability to traverse biological barriers. However, it is important to note that these rules are not absolute, and exceptions exist. Some compounds that do not strictly adhere to these guidelines can still exhibit good bioavailability and bioactivity with appropriate optimization or formulation strategies. Despite their limitations, empirical rules remain essential tools in drug discovery, providing valuable insights into the physicochemical properties that contribute to a molecule’s potential bioactivity.

Bioactivity is a key property of molecules that determines their potential to interact with biological systems and elicit a pharmacological response. Likewise, in this step of the drug development studies, in silico QSAR models can be of great helpful. Models that are more directed towards the bioactive properties of the compounds use mathematical models of structure–activity correlation, and that are able to predict bioactivity related properties, such as, Absorption, Distribution, Metabolism, Excretion, and Toxicity (ADMET), can be very useful instruments in a second step of selection of the most promising bioactives. In silico ADMET tools [148,155,156] have become increasingly essential in drug discovery, as they enable the rapid prediction of a compound’s pharmacokinetics and safety profile. By providing such information, these tools allow researchers to evaluate and optimize candidate molecules early in the drug development process, and consequently, reduce attrition rates, minimize the need for costly animal testing, and expedite the translation of novel compounds from bench to bedside.

Other powerful tools to facilitate/speed up the drug development process are the SwissTargetPrediction [157,158] and SEA [159,160] online molecule-ligand search services, which identify the most likely targets for the studied compound.

On the other hand, online molecular docking services (e.g., swissDOCK) [161] facilitate the identification of potential drug targets and elucidate the binding modes of drug molecules to their corresponding receptors. By simulating the molecular interactions between ligands and proteins, these services provide valuable information on the affinity, selectivity, and stability of drug–receptor complexes. These data are crucial in guiding the rational design of novel therapeutic agents and optimizing lead compounds for improved efficacy and safety.

However, in silico methods, while powerful, have limitations. Their accuracy depends on the quality of input data and algorithms used. Incomplete or imprecise structural information can lead to incorrect predictions. Additionally, these methods may struggle to capture dynamic protein–ligand interactions or fully account for the complex, multifactorial nature of ADMET properties. Furthermore, computational power and algorithm efficiency can limit the exploration of large chemical spaces, and the inherent bias in training datasets may affect the generalizability of the predictions.

Finally, for each of the above-presented molecules (ergosterol, octacosanol, N-palmitoylsphingomyelin, α-Tocopherol, phosphatidylcholine(16:0/20:5), phosphatidylcholine (16:0/22:6), butyrate and sodium butyrate), the parameters obtained through these in silico models, and whether that information aligns with what has been previously discussed for each one of them, will be commented on. The research on in silico QSAR models was conducted using the respective SMILES of each molecule (Table S2).

### 4.1. Ergosterol

Ergosterol is characterized by PubChem and ChemSpider with a molecular weight of 396.51 g/mol, a MlogP around 6.33/7.18 [150,162] (depending on the source), and a low solubility in water (2.84 × 10^−4^ mg/mL [139] being the highest value accessed) (Table 2). While these results indicate its high lipophilicity (above the optimal range, Table 1), it also supports the fact that ergosterol is considered a good drug candidate since it is accepted by Lipinski and Veber druglikeness rules (Table S3).

Furthermore, the ergosterol’s TPSA value of 20.19 Å^2^, is indicative of both good intestinal absorption (i.e., <120 Å^2^) and good BBB crossing (<90 Å^2^). Even so, regarding these properties, some QSAR databases are in disagreement (Table 3); while SwissADME states that ergosterol has a low Human Intestinal Absorption (HIA) and is not able to penetrate the BBB, pkCSM indicates a 95.4% HIA and a logBB of 0.76 (meaning it penetrates the BBB readily ([163])).

None of the following parameters were found to influence the ergosterol’s druglikeness: Pan Assay Interference compounds (PAINS), Alarm NMR Rule (associated with thiol-reactive compounds), BMS Rule based on reactive compounds, and Chelator Rule (chelating compounds) (Table S4). Nevertheless, the Rat Oral Acute Toxicity (LD50) was 2.26 mol/Kg and the Maximum Human Tolerated Daily Dose was equal to 0.20 mg/Kg/day (Table 3).

The SwissTargetPrediction identifies ergosterol as a Vitamin D receptor (VDR) (Table S5) and, consequently, a nuclear receptor of calcitriol, the active form of vitamin D_3_. Both SwissTargetPrediction and SEA predictions described ergosterol’s correlation to cholesterol biosynthesis, its cellular protection from oxidative stress, and identified it as the direct molecular target of ezetimibe, a drug that inhibits cholesterol absorption that is approved for the treatment of hypercholesterolemia. The QSAR generated information that can be a complement to the previously described in vitro and in vivo evidence on ergosterol anti-obese properties. Nevertheless, a comparison of bibliographic information on the mode of action of ergosterol with in silico reviewed parameters showed other distinguishable results. Hence, ergosterol activity on steroid 17-alpha-hydroxylase involved in corticoid and androgen biosynthesis, as well as the correlation of ergosterol with Glycine Receptor Subunit Alpha-1 (GLRA1), that play a major role in mediating fast inhibitory neurotransmission in the spinal cord and brain stem, were not discussed in this literature review, but generated outputs in QSAR prediction.

### 4.2. Policosanol

As previously mentioned, policosanol is a mixture of fatty alcohols, octacosanol being the main constituent, and, consequentely, in silico parameters were generated indicating octacosanol SMILES.

Octacosanol molecular weight is 410.66 g/mol, its MlogP is about 7.07/9.83 [150,162] (according to SwissADME and Molinspiration, respectively), and it presents a low solubility in water (in the 1 × 10^−7^ mg/mL order) (Table 2). Despite its high lipophilicity (above optimal range, Table 1), its TPSA value, 20.19 Å^2^ (Table 2) indicates that this molecule should have a good HIA and BBB penetration performance. Hence, different QSAR models state different results towards the mentioned properties (Table 3); SwissADME suggests a low HIA and a lack of BBB penetration capacity, while pkCSM predicts a HIA of 85.7% and a logBB value of 1.03 (indicating high BBB penetration [163].

The druglikeness parameters of octacosanol states that the molecule is accepted as a good drug candidate according to Lipinski’s rule of five, but not for any of the other addressed methodologies (Table S3). Furthermore, a BMS Rule (based on reactive compounds) violation was noted (Table S3) and the results for ADMET properties, such as Rat Oral Acute Toxicity (LD50), was 1.87 mol/Kg and the Maximum Human Tolerated Daily Dose was 0.40 mg/Kg/day (Table 3).

SEA target prediction (Table S5) suggests that octacosanol catalyses the NAD-dependent oxidation of all-trans-retinol, alcohol, and omega-hydroxy FAs and their derivatives, which is in accordance with the previously discussed association of octacosanol to the β-oxidation of FAs by Kabir et al. [31]. Contrary to the above-discussed bibliographic information, QSAR prediction also relates octacosanol influence in vesicular trafficking processes and in receptor-mediated endocytosis, as well as in the catalysation of IPP addition onto DMAPP to form geranylgeranyl pyrophosphate, an important precursor of carotenoids and geranylated proteins, according to SEA. Furthermore, SwissTargetPrediction predicted that octacosanol binds to a receptor-activated non-selective cation channel involved in the detection of sensations such as coolness (cold temperature).

### 4.3. Dietary Sphingomyelin

For QSAR analysis, the SM from chicken egg (N-Palmitoylsphingomyelin; d18:1/16:0) was used as reference, since is the most described in the literature, not only in terms of bioactive properties, but also regarding the SM mechanism of action. This molecule possesses a molecular weight of 702.90 g/mol and a logP > 5.5 (very lipophilic) (Table 2). According to the Lipinski rule, this SM respects the druglikeness parameters, but not according to all other five rules considered (Table S3). Prediction models found no interfering, thiol reactive, or chelating compound alerts, just one reactive compound alert according to BMS rule (Table S3). The SwissADME model attributed a low score for HIA, while the pkCSM model predicted that 79.2% of the SM would be absorbed (Table 3). The egg SM bioavailability score stands at 0.55 (Table S3), while the fraction unbound in plasm is 0.21 Fu (Table 3), this lipid having a good cell permeation (TPSA < 120 A^2^; Table 2). This SM might constitute a substrate of intestinal P-glycoprotein, but not of renal OCT2 (Table 3), which are transport proteins involved in the excretion of xenobiotics [164]. Moreover, models predicted that egg SM is hepatoxic to humans, and that no more than 0.74 mg/Kg/day should be ingested (Table 3).

Among the targets suggested by prediction models (Table S5), egg SM has been described to inhibit secretory (type II) phospholipase A2 (PLA2; Protein Data Bank ID—5G3N) [165,166], which is structurally similar to pancreatic (type I) PLA2 (Protein Data Bank ID—6Q42), with a TM-score [0, 1] = 0.86, according to RCSB Protein Data Bank (www.rcsb.org; [167]). Pancreatic PLA2 catalyses the release of FAs and lysophospholipids, principally LPC, from PLs during digestion [168]. The LPC has shown to be important in cholesterol intestinal absorption process [169]. Thus, it has been proposed that SM might decrease cholesterol uptake through the inhibition of pancreatic PLA2 [13]. However, this assumption was based on the SM inhibitory effect towards another PLA2, as stated initially. Therefore, the dietary SM modulation of pancreatic PLA2 activity, and its subsequent impact in cholesterol absorption, would need to be studied to confirm the proposed way-of-action of SM in cholesterol homeostasis.

On the other hand, increased secretory (type II) PLA2 has been associated with elevated cardiovascular event risk in patients with cardiovascular or metabolic disorders [170,171]. Therefore, SM treatment could possibly prevent cardiovascular event through the inhibition of secretory (type II) PLA2; however, this enzyme has not been well established as a therapeutic target in such health conditions yet. Despite the possible association, when compared to what is described in the literature, these targets do not represent the principal stakeholders in dietary SM activity against dyslipidaemia.

### 4.4. α-Tocopherol

Regarding the physicochemical properties of this molecule (Table 4), α-Toc has a molecular weight of 430.59 g/mol (therefore under the 500 g/mol cut-off) and TPSA value of 29.40 Å^2^, pointing out good intestinal and brain absorption. However, the lipophilicity indicators (i.e., 9–12 for XlogP and 8.84 for WlogP) suggest that this molecule is highly lipophilic (far above the optimal range), which may impair its absorption. Even when considering the effect of pH (i.e., logD), the values are above the limit. Still, α-Toc is approved by the Lipinski rule of five, although it does not match the other drugability methodologies addressed in this work. ADMET properties (Table 5) show Caco-2 absorption (Log Papp 1.35) and its capacity to diffuse through the BBB (log BB value of 0.88), which is in agreement with the existing literature in which α-TTP has been described as being responsible for α-Toc uptake into the cell, and ABCA1 as responsible for excretion and uploading into chylomicron remnants (when transported to the liver) and other lipoproteins (for transportation in the blood). Those transporters have been described as being expressed in different cells in the brain, while, in the BBB, SRB1 will perform the transportation of this molecule. However, the target prediction results (Table S6) show a hit for α-TTP.

### 4.5. Glycerophosphatidylcholines from Krill Oil

Both molecules appear in the literature as potential bioactives with the ability to restore brain biochemistry, at least in relation to cholesterol homeostasis. However, descriptors obtained from different in silico online services (please see and Table 1, Table 4, Table 5, Tables S4 and S6 for further details) show that these two molecules may have some drawbacks.

As shown in Table 4, the molecular weight for both molecules is 779.93 g/mol for PC (16:0/20:5) and 805.95 g/mol for PC (16:0/22:6). This initially indicates that the molecules are too large (MW cut-off of 500 g/mol) and may have difficulty in being absorbed. On the other hand, the values of TPSA (111/121 for both cases) fall within the optimal range for intestinal absorption (i.e., <120 Å^2^) but above the defined value for crossing the BBB (<90 Å^2^). The lipophilicity values (i.e., XlogP) are also found to be above the optimal value (11.90 and 12.50, respectively) (Table 1). According to the BOILED-egg model [152], the value of WlogP, for these molecules to cross the BBB, should be less than 6 and greater than 1.5, being 12.12 and 12.68 for the two molecules. However, the logD values are below three in both cases.

Considering the consulted literature, for these compounds to exert their action, they first need to cross the BBB through interaction with MFSD2A, which will transport them into the brain. The BOILED-egg model assumes diffusion, and not transportation, through a receptor, which may explain the obtained result.

Once inside the brain, they must bind to either TREM2 (to promote myelin debris removal) or with ABCA1 and ABCG1 to be incorporated into nascent ApoE particles. In this sense, both PCs do not need to cross the cell membrane but rather reach it. This seems possible either by the already discussed mechanism of intestinal absorption or by the TPSA values.

None of these targets (i.e., MFSD2A, TREM2, ABCA1, and ABCG1) were identified by the consulted online services. This may be because i) the models must have the structures of these receptors in their databases, or ii) organs or tissues cannot be selected in these services, thus the model provides a general answer. Although they represent a great help in determining possible bioactivities, in this case, it becomes evident that a specific approach, using direct simulation (i.e., docking) between the ligand and the protein, would give a more adequate response.

### 4.6. Butyrate and Sodium Butyrate

Butyrate and NaB are organic compounds with similar physicochemical properties. According to PubChem, the molecular weight of butyrate is 88.09 g/mol and 110.08 g/mol for NaB (Table 4). Hence, as these molecular weights are lower than 500 g/mL (Table 1) these molecules can be easily absorbed and cross cell membranes. The TPSA is similar for both compounds—37.20 Å^2^ for butyrate and 40.13 Å^2^ for NaB—indicating, again, that these molecules are more likely to cross biological membranes, since the values are <140 Å^2^. The low XlogP value (0.79) indicates that both molecules are hydrophilic; in fact, butyrate´s water solubility is 0.16 mg/mL and NaB is 14.20 mg/mL. These physicochemical characteristics can affect its ADMET properties. As shown in Table 5, the values for log Papp Caco-2 permeability for butyrate is 1.56 and 1.45 for NaB. These values indicate good intestinal absorption since they are higher than 0.9 [172].

Moreover, as predicted by SwissADME both molecules show high HIA, and, according to the pKCSM platform, HIA for butyrate and NaB is 92.2% and 100%, respectively. The pkCSM platform also indicates that both molecules can penetrate the BBB (values of logBB are higher than -1 for both molecules [163]). Regarding the metabolism prediction, nor butyrate nor NaB are likely to be cytochrome P450 inhibitors or metabolized by P450. The prediction models indicate that neither of the molecules are likely to be a substrate for the renal transporter (OCT2, excretion parameter) nor mutagenic (Ames Toxicity negative, toxicity parameter).

When QSAR models were applied to predict the receptors involved in butyrate and NaB, using SwissTargetPrediction and SEA, only the HDAC receptor and GABA transporter matched with the experimental research work (Table S6).

However, it is important to note that QSAR models are as good as the data used to build them and are not a replacement for experimental validation. Their predictions should always be verified through laboratory experiments.

## 5. Conclusions

The current review deepens and bring together evidence regarding how lipids are involved in different homeostasis processes, focusing on those related to obesity and brain biochemistry, by their interaction with molecular targets as, for example, membrane receptors, nuclear factors, and lipoproteins, among others. Thus, it has been possible to summarize the state-of-the art related to the markers associated with the hypocholesterolemic activity of ergosterol, policosanol, and dietary SM. There is a full set of data suggesting that these molecules may interfere in hepatic lipogenesis pathways and therefore control cholesterol homeostasis by reducing its absorption. Hence, some bioactivities, that were not found in the literature review section, were generated as outputs from QSAR prediction (i.e., ergosterol role in corticoid and androgen biosynthesis and in mediating fast inhibitory neurotransmission; octacosanol influence in vesicular trafficking and endocytosis processes, and SM correlation to ovarian cancer).

Alpha-Tocopherol was shown to prevent depression, likely due to its antioxidant properties, and some α-Toc in silico results agreed with the existing literature, where the prediction results indicated α-TTP as α-Toc target.

Glycerophosphatidylcholines are described in the literature as potential molecules that contribute to normal brain function (identified targets: MFSD2A, TREM2, ABCA1, and ABCG1). Nevertheless, the consulted in silico models failed to highlight those molecular targets.

Also, in this work, several studies evidenced the potential of butyrate and NaB on NDs essentially by their modulatory effect of the inflammatory response (e.g., GPR109A, NF-kB, or IL-6). Computationally generated targets, such as HDAC receptor and GABA transporter, appeared on both the revised experimental works and QSAR analysis.

However, the present study found that the targets obtained by computational models are often not consistent with the existing evidence from in vivo and in vitro experimental studies. Accordingly, more research is necessary to provide information on effective drug dose, as well as pharmacokinetics and pharmacodynamics, to improve the predictiveness of the existing QSAR, or for new ones to be developed.

## Figures and Tables

**Table 1:** Drugability methodologies parameters.

Drugability Methodologies	MW (g/mol)	logP	#N or O (H-Bond Acceptors)	#NH or OH (H-Bond Donors)	TPSA (Å^2^)	# Atoms	# Carbons	# Heteroatoms	# Rotatable Bonds	# Rings	MR (m^3^.mol^−1^)
**Lipinski**	≤500	MlogP ≤ 4.15	≤10	≤5	n.d	n.d	n.d	n.d	n.d	n.d	n.d
**Ghose**	160 to 480	WlogP: −0.4 to 5.6	n.d	n.d	n.d	20 to 70	n.d	n.d	n.d	n.d	40 to 130
**Veber**	n.d	n.d	n.d	n.d	≤140	n.d	n.d	n.d	≤10	n.d	n.d
**Egan**	n.d	WlogP: ≤5.88	n.d	n.d	≤131.6	n.d	n.d	n.d	n.d	n.d	n.d
**Muegge**	200 to 600	XlogP: −2 to 5	≤10	≤5	≤150	n.d	>4	>1	≤15	≤7	n.d
**Leadlikeness**	250 to 350	XlogP ≤3.5	n.d	n.d	n.d	n.d	n.d	n.d	≤7	n.d	n.d

n.d—not defined; the parameter was not considered/defined by the empirical model.

**Table 2:** Physico-chemical properties of Ergosterol, Octacosanol and N-Palmitoylsphingomyelin.

Physico-Chemical Properties	Ergosterol	Octacosanol	N-Palmitoylsphingomyelin
MW (g/mol)	396.51	410.66	702.90
TPSA (Å^2^)	20.19	20.19	108 ^abcd^/118 ^ef^
XlogP	7.40	13.68	10 ^deh^/12 ^af^
WlogP	7.33	10.14	10.90
MlogP	6.33 ^f^/7.18 ^b^	7.07 ^f^/9.83 ^b^	5.54
# H-Bond Donors	1	1	2 ^abcdfh^/3 ^e^
# H-Bond Acceptors	1	1	6 ^adfh^/8 ^bce^
# Rotatable Bonds	4	26	36 ^abdeh^/37 ^cf^
# Rings	4	0	0
# Atoms	73	87	48
# Heteroatoms	1	1	9
# Carbons	28	28	39
Molar Refractivity (MR) (m^3^.mol^−1^)	124.2 ^e^/127.5 ^f^	133.3 ^e^/137.9 ^f^	206.11
Melting Point (°C)	150 ^g^/157 ^e^/170 ^a^	83 ^a^/98 ^g^	190
Volume (cm^3^)	394.1 ^e^/427.4 ^b^/459.5 ^ci^	488.2 ^e^/501.6 ^ci^	750.98 ^b^/772.39 ^c^
Density (g/cm^3^)	1.00	0.80	0.91
logD (pH 7.4)	5.67 ^ci^/7.99 ^e^	4.56	3.05 ^c^/8.27 ^e^
logS (water)	−6.55	−9.3 ^f^/−7.5 ^c^/−6.9 ^h^	−2.9 ^c^/−4.0 ^h^/−7.2 ^g^/−9.5 ^f^
Water Solubility (mg/mL)	4.3 × 10^−5 a^/7.6 × 10^−5 f^/2.8 × 10^−4 e^	1.40 × 10^−7 ae^/2.34 × 10^−7 f^	2.01 × 10^−7^
pKa	13.90	13.90	1.7 OH/12.0 NH

a—https://pubchem.ncbi.nlm.nih.gov/; b—https://www.molinspiration.com/; c—https://admetmesh.scbdd.com/; d—https://xundrug.cn/molgpka; e—https://www.chemspider.com/; f—http://www.swissadme.ch/; g—https://ochem.eu/home/show.do; h—http://biosig.unimelb.edu.au/pkcsm/; i—https://bidd.group/NPASS/index.php. Accessed on 18 January 2023.

**Table 3:** ADMET properties of Ergosterol, Octacosanol and N-Palmitoylsphingomyelin.

ADMET Properties	Ergosterol	Octacosanol	N-Palmitoylsphingomyelin
Absorption			
Caco2 permeability (log Papp in 10^−6^ cm/s)	1.22	1.08	0.49
P-glycoprotein I inhibitor (Yes/No)	Yes	No	No
P-glycoprotein II inhibitor (Yes/No)	Yes	Yes	Yes
P-glycoprotein (Pgp) substrate (Yes/No)	No	Yes ^a^/No ^b^	Yes
Human intestinal absorption (HIA) (% Absorbed)	Low ^a^/95.4 ^b^	Low ^a^/85.7 ^b^	Low ^a^/79.2 ^b^
log Kp (cm/s)	−3.44 ^a^/−2.81 ^b^	0.86 ^a^/−2.74 ^b^	−1.81 ^a^/−2.74 ^b^
Distribution			
Blood–Brain Barrier (BBB) Penetration (log BB)	No ^a^/0.76 ^b^	No ^a^/1.03 ^b^	No ^a^/−1.74 ^b^
Volume Distribution (log L/Kg)	0.27	0.02	−0.70
Fraction unbound in plasms (Fu)	0	0.07	0.21
Metabolism			
Cytochrome P450 1A2 inhibitor (CYP1A2) (Yes/No)	No	Yes ^c^/No ^ab^	No
Cytochrome P450 2C19 inhibitor (CYP2C19) (Yes/No)	No	No	No
Cytochrome P450 2C9 inhibitor (CYP2C9) (Yes/No)	Yes^a^/No ^bc^	No	No
Cytochrome P450 2D6 inhibitor (CYP2D6) (Yes/No)	No	Yes^c^/No ^ab^	No
Cytochrome P450 2D6 substrate (CYP2D6) (Yes/No)	No	No	No
Cytochrome P450 3A4 inhibitor (CYP3A4) (Yes/No)	No	No	Yes ^a^/No ^bc^
Cytochrome P450 3A4 substrate (CYP3A4) (Yes/No)	Yes	Yes	Yes
Excretion			
Total Clearance (log mL/min/Kg)	0.56	2.15	1.35
Renal OCT2 substrate (Yes/No)	No	No	No
Toxicity			
hERG I inhibitor (Yes/No)	No	No	No
hERG II inhibitor (Yes/No)	Yes	Yes	No
Human Hepatotoxicity (Yes/No)	No	No	Yes
AMES Toxicity (Yes/No)	No	No	No
Rat Oral Acute Toxicity (LD50) (mol/Kg)	2.26	1.87	2.71
Maximum Tolerated Daily Dose (human) (log mg/Kg/day)	−0.69	−0.40	−0.13
Skin Sensitization	No	Yes ^b^/No ^de^	Yes ^d^/No ^be^

a—http://www.swissadme.ch/; b—http://biosig.unimelb.edu.au/pkcsm/; c—https://ochem.eu/home/show.do; d—http://predskin.labmol.com.br/; e—https://nerdd.univie.ac.at/skinDoctorII/. Accessed on 18 January 2023.

**Table 4:** Physico-chemical properties of α-Tocopherol, Phosphatidylcholines (16:0/20:5) and (16:0/22:6), Butyrate, and Sodium Butyrate.

Physico-Chemical Properties	α-Tocopherol (Vitamin E)	Phosphatidylcholine (16:0/20:5)	Phosphatidylcholine (16:0/22:6)	Butyrate	Sodium Butyrate
MW (g/mol)	430.59	779.93	805.95	88.09	110.08
TPSA (Å^2^)	29.40	111 ^abcd^/121 ^ef^	111 ^abcd^/121 ^ef^	37.20	40.13
XlogP	9 ^gh^/10 ^i^/11 ^f^/12 ^ae^	11.90	12.50	0.79	0.79
WlogP	8.84	12.12	12.68	0.87	−0.46
MlogP	6.14 ^f^/9.04 ^b^	---	---	0.49 ^f^/1 ^b^	−1.72 ^b^/0.49 ^f^
# H-Bond Donors	1	0 ^abcdfh^/1 ^e^	0 ^abcdfh^/1 ^e^	1	0
# H-Bond Acceptors	2	8 ^adfh^/9 ^bce^	8 ^adfh^/9 ^bce^	2	2
# Rotatable Bonds	12	37 ^dh^/39 ^abcef^	38 ^dh^/40 ^abcef^	2	2
# Rings	2	0	0	0	0
# Atoms	31	132	136	6	6
# Heteroatoms	2	10	10	2	3
# Carbons	29	44	46	4	4
Molar Refractivity (MR) (m^3^.mol^−1^)	135.1 ^e^/139.3 ^f^	226.66	235.80	22.61	21.17
Melting Point (°C)	2 ^e^/3 ^a^	149.70	160.00	−5.35	250–253
Volume (cm^3^)	462.8 ^e^/474.5 ^b^/502.7 ^ci^	809.94 ^b^/852.27 ^c^	884.23	89.2 ^e^/89.8 ^b^/92.7 ^ci^	87.1 ^b^/91.4 ^c^
Density (g/cm^3^)	0.90	0.92	0.91	0.96	0.95
logD (pH 7.4)	6.9 ^ci^/10.3 ^e^	2.37 ^c^/8.32 ^e^	2.26 ^c^/8.94 ^e^	−1.75 ^e^/1.10 ^ci^	−1.23
logS (water)	−7.0 ^cih^/−7.8 ^g^/−8.6 ^f^	−3.42 ^h^/−2.39 ^c^	−3.29 ^h^/−2.04 ^c^	−0.75 ^f^/−0.19 ^ah^/0.11 ^ci^	−0.89 ^f^/0.25 ^h^/0.69 ^c^
Water Solubility (mg/mL)	---	4.10 × 10^−5^	3.90 × 10^−5^	0.16	14.20
pKa	10.80	---	---	4.76	---

a—https://pubchem.ncbi.nlm.nih.gov/; b—https://www.molinspiration.com/; c—https://admetmesh.scbdd.com/; d—https://xundrug.cn/molgpka; e—https://www.chemspider.com/; f—http://www.swissadme.ch/; g—https://ochem.eu/home/show.do; h—http://biosig.unimelb.edu.au/pkcsm/; i—https://bidd.group/NPASS/index.php. Accessed on 18 January 2023.

**Table 5:** ADMET properties of α-Tocopherol, Phosphatidylcholines (16:0/20:5) and (16:0/22:6), Butyrate, and Sodium Butyrate.

ADMET Properties	α-Tocopherol (Vitamin E)	Phosphatidylcholine (16:0/20:5)	Phosphatidylcholine (16:0/22:6)	Butyrate	Sodium Butyrate
Absorption					
Caco2 permeability (log Papp in 10^−6^ cm/s)	1.35	0.65	0.65	1.56	1.45
P-glycoprotein I inhibitor (Yes/No)	No	No	No	No	No
P-glycoprotein II inhibitor (Yes/No)	Yes	Yes	Yes	No	No
P-glycoprotein (Pgp) substrate (Yes/No)	Yes ^a^/No ^b^	Yes ^a^/No ^b^	Yes ^a^/No ^b^	No	Yes
Human intestinal absorption (HIA) (% Absorbed)	Low ^a^/89.8 ^b^	Low ^a^/99.7 ^b^	Low ^a^/100 ^b^	High ^a^/92.2 ^b^	High ^a^/100 ^b^
log Kp (cm/s)	−1.33 ^a^/−2.68 ^b^	−2.52 ^a^/−2.74 ^b^	−2.26 ^a^/−2.73 ^b^	−6.28 ^a^/−2.75 ^b^	−6.41 ^a^/−2.76 ^b^
Distribution					
Blood-Brain Barrier (BBB) Penetration (log BB)	NA ^a^/0.88 ^b^	NA ^a^/−2.09 ^b^	NA ^a^/−2.10 ^b^	Yes ^a^/−0.26 ^b^	No ^a^/−0.25 ^b^
Volume Distribution (log L/Kg)	0.71	−1.18	−1.27	−0.83	−0.88
Fraction unbound in plasms (Fu)	0	0.21	0.23	0.69	0.83
Metabolism					
Cytochrome P450 1A2 inhibitor (CYP1A2) (Yes/No)	No	No	No	No	No
Cytochrome P450 2C19 inhibitor (CYP2C19) (Yes/No)	Yes ^b^/No ^ac^	No	No	No	No
Cytochrome P450 2C9 inhibitor (CYP2C9) (Yes/No)	No	Yes ^a^/No ^bc^	Yes ^a^/No ^bc^	No	No
Cytochrome P450 2D6 inhibitor (CYP2D6) (Yes/No)	No	No	No	No	No
Cytochrome P450 2D6 substrate (CYP2D6) (Yes/No)	No	No	No	No	No
Cytochrome P450 3A4 inhibitor (CYP3A4) (Yes/No)	No	No	No	No	No
Cytochrome P450 3A4 substrate (CYP3A4) (Yes/No)	Yes	Yes	Yes	No	No
Excretion					
Total Clearance (log mL/min/Kg)	0.79	1.03	0.96	0.42	1.10
Renal OCT2 substrate (Yes/No)	No	No	No	No	No
Toxicity					
hERG I inhibitor (Yes/No)	No	No	No	No	No
hERG II inhibitor (Yes/No)	Yes	No	No	No	No
Human Hepatotoxicity (Yes/No)	No	No	No	No	No
AMES Toxicity (Yes/No)	No	No	No	No	No
Rat Oral Acute Toxicity (LD50) (mol/Kg)	2.07	2.53	2.56	1.72	1.26
Maximum Tolerated Daily Dose (human) (log mg/Kg/day)	0.78	0.36	0.36	0.91	0.71
Skin Sensitization	Yes ^de^/No ^b^	Yes ^d^/No ^be^	Yes ^d^/No ^be^	No	No

a—http://www.swissadme.ch/; b—http://biosig.unimelb.edu.au/pkcsm/; c—https://ochem.eu/home/show.do; d—http://predskin.labmol.com.br/; e—https://nerdd.univie.ac.at/skinDoctorII/; Accessed on 18 January 2023. NA—not available.

## Data Availability

Not applicable.

## Supplementary Materials

The following supporting information can be downloaded at: https://www.mdpi.com/article/10.3390/foods12132576/s1, Table S1: Tocopherol content of various plant food sources; Table S2: Identification of the studied molecules; Table S3: Druglikeness results for Ergosterol, Octacosanol and N-Palmitoylshingomyelin; Table S4: Druglikeness results for α-Tocopherol, Phosphatidylcholines (16:0/20:5) and (16:0/22:6), Butyrate and Sodium Butyrate; Table S5: Target prediction for Ergosterol, Octacosanol and N-Palmitoylshingomyelin; Table S6: Target prediction α-Tocopherol, Phosphatidylcholines (16:0/20:5) and (16:0/22:6), Butyrate and Sodium Butyrate.

## Abbreviations

AD—Alzheimer´s disease; ADMET—Absorption, Distribution, Metabolism, Excretion, Toxicity; ApoE—Apolipoprotein E; α-Toc—Alpha-Tocopherol; α-TTP—Alpha-Tocopherol transfer protein; Aβ—Beta-amyloid protein; BA—Bile acid; BBB—Blood–brain barrier; CNS—Central nervous system; FA—Fatty acid; FAS—Fatty acid synthase; GABA—Gamma-aminobutyric acid; GPR—G protein-coupled receptor; HDAC—Histone deacetylase; HDL—High-density lipoprotein; HFD—High-fat diet; HIA—Human intestinal absorption; HMG-CoA—3-hidroxi-3-methyl-glutaril-CoA reductase; IL—Interleukin; LDL—Low-density lipoprotein; LDL-R—Low-density lipoprotein-receptor; LPC—Lysophosphatidylcholine; MFSD2A—Major facilitator superfamily domain-containing protein 2; NaB—Sodium butyrate; NAFLD—Non-alcoholic fatty liver disease; ND—Neurodegenerative disease; PC—Phosphatidylcholine; PD—Parkinson´s disease; PL—Phospholipid; PPAR-γ—Peroxisome proliferator-activated receptor-gamma; QSAR—Quantitative Structure–Activity Relationship; SM—Sphingomyelin; SMILES—Simplified Molecular-Input Line-Entry System; SRB1—Scavenger receptor class B type 1; SREBP—Sterol regulatory element-binding protein; TG—Triglyceride; TNF-α—Tumour necrosis factor-alpha; TPSA—Topological surface area; TREM2—Triggering receptor expressed on myeloid cells 2.
