# Supplementary material for: Novel Lipids to Regulate Obesity and Brain Function: Comparing Available Evidence and Insights from QSAR In Silico Models
Source: Foods. 2023 Jul 1;12(13):2576. doi: 10.3390/foods12132576 (PMC10341324; doi:10.3390/foods12132576)
Supplement: Supplementary file 1 [file foods-12-02576-s001.zip › foods-2410062-supplementary.pdf]

foods

*Table S1.* Tocopherol content of various plant food sources.

| Food source | α-Tocopherol (mg/100 g) | β-Tocopherol (mg/100 g) | γ-Tocopherol (mg/100 g) | δ-Tocopherol (mg/100 g) |
|---|---|---|---|---|
| **Apricot oil** | 1.3 - 3.1 | - | 66.4 - 81.0 | 1.7 - 2.5 |
| **Hemp seed oil** | 3.1 | - | 60 | 3 |
| **Safflower oil** | 57 - 130 | - | 1.4 - 2.6 | 0 - 0.70 |
| **Sunflower oil** | 51 | - | 2.2 | 0.5 |
| **Almond** | 22 - 30 | - | - | - |
| **Hazelnut raw** | 26 - 32 | - | 2.3 - 3.9 | 0.2 - 0.4 |
| **Peanut** | 7.4 - 13 | 0.2 - 0.5 | 8.7 - 23 | 0.4 - 1 |
| **Walnut** | 2.8 - 4 | - | 16 - 31 | 1.7 - 4 |

**Contents according to Zaaboul et al. [68]**

foods MDPI

*Table S2.* Identification of the studied molecules.

| Name | SMILES |
|---|---|
| **Ergosterol** | C[C@H](/C=C/[C@H](C)C(C)C)[C@H]1CC[C@@H]2[C@@]1(CC[C@H]3C2=CC=C4[C@@]3(CC[C@@H](C4)O)C)C |
| **Octacosanol** | CCCCCCCCCCCCCCCCCCCCCCCCCCCCO |
| **N-Palmitoylsphingomyelin** | [H][C@](/C=C/CCCCCCCCCCCCC)(O)[C@@]([H])(NC(CCCCCCCCCCCCCCC)=O)COP([O-])(OCC[N+](C)(C)C)=O |
| **α-Tocopherol (Vitamin E)** | CC1=C(C2=C(CC[C@@](O2)(C)CCC[C@H](C)CCC[C@H](C)CCCC(C)C)C(=C1O)C)C |
| **Phosphatidylcholine(16:0/20:5)** | CCCCCCCCCCCCCCCC(=O)OCC(COP(=O)([O-])OCC[N+](C)(C)C)OC(=O)CCCC=CCC=CCC=CCC=CCC=CCC |
| **Phosphatidylcholine (16:0/22:6)** | CCCCCCCCCCCCCCCC(=O)OCC(COP(=O)([O-])OCC[N+](C)(C)C)OC(=O)CCC=CCC=CCC=CCC=CCC=CCC=CCC |
| **Butyrate** | CCCC(=O)O |
| **Sodium Butyrate** | CCCC(=O)[O-].[Na+] |

***Table S3.*** Druglikeness results for Ergosterol, Octacosanol and N-Palmitoylshingomyelin.

| Druglikeness Parameters | Ergosterol | Octacosanol | N-Palmitoylsphingomyelin |
|---|---|---|---|
| **Lipinski (# violations; which)** | 1 (MlogP) | 1 (MlogP) | 1 (MW) |
| **Lipinski Acceptance** | Yes | Yes | Yes |
| **Ghose (# violations; which)** | 2 (WlogP; MR) | 3 (WlogP; #Atoms; MR) | 4 (MW; WlogP; #Atoms; MR) |
| **Ghose Acceptance** | No | No | No |
| **Veber (# violations; which)** | 0 | 1 (#Rotatable Bonds) | 1 (#Rotatable Bonds) |
| **Veber Acceptance** | Yes | No | No |
| **Egan (# violations; which)** | 1 (WlogP) | 1 (WlogP) | 1 (WlogP) |
| **Egan Acceptance** | No | No | No |
| **Muegge (# violations; which)** | 2 (XlogP; #Heteroatoms) | 3 (XlogP; #Heteroatoms; #Rotatable Bonds) | 3 (XlogP; #Heteroatoms; #Rotatable Bonds) |
| **Muegge Acceptance** | No | No | No |
| **Leadlikeness (# violations; which)** | 2 (MW; XlogP) | 3 (MW; XlogP; #Rotatable Bonds) | 3 (MW; XlogP; #Rotatable Bonds) |
| **Leadlikeness Acceptance** | No | No | No |
| **Bioavailability score** | 0.55 | 0.55 | 0.55 |
| **Synthetic accessibility score (SAscore)** | 4.63 | 1.49[bc] / 3.72[a] | 4.24[b] / 7.82[a] |
| **Natural product-likeness score (Npscore)** | 3.04 | 0.26 | 0.66 |
| **Fsp3** | 0.79 | 1 | 0.92 |
| **Brenk (Structural alert)** | 1 | 0 | 3 |
| **Medicinal Chemistry Evaluation (MCE-18)** | 68.04 | 0 | 9 |
| **PAINS (Pan Assay Interference compounds)** | 0 | 0 | 0 |
| **Alarm NMR Rule (Thiol reactive compounds)** | 0 | 0 | 0 |
| **BMS Rule (Reactive compounds)** | 0 | 1 | 1 |
| **Chelator Rule (Chelating compounds)** | 0 | 0 | 0 |
| **QED (Desirability)** | 0.49 | 0.14 | 0.03 |

a - http://www.swissadme.ch/; b - https://admetmesh.scbdd.com/; c - https://bidd.group/NPASS/index.php

***Table S4.*** Druglikeness results for $\alpha$-Tocopherol, Phosphatidylcholines (16:0/20:5) and (16:0/22:6), Butyrate and Sodium Butyrate.

| **Druglikeness Parameters** | **$\alpha$-Tocopherol (Vitamin E)** | **Phosphatidylcholine (16:0/20:5)** | **Phosphatidylcholine (16:0/22:6)** | **Butyrate** | **Sodium Butyrate** |
|---|---|---|---|---|---|
| **Lipinski (# violations; which)** | 1 (MlogP) | 1 (MW) | 1 (MW) | 0 | 0 |
| **Lipinski Acceptance** | Yes | Yes | Yes | Yes | Yes |
| **Ghose (# violations; which)** | 2 (WlogP; MR) | 3 (MW; #Atoms; MR) | 3 (MW; #Atoms; MR) | 3 (MW; #Atoms; MR) | 4 (MW; WlogP; #Atoms; MR) |
| **Ghose Acceptance** | No | No | No | No | No |
| **Veber (# violations; which)** | 1 (#Rotatable Bonds) | 1 (#Rotatable Bonds) | 1 (#Rotatable Bonds) | 0 | 0 |
| **Veber Acceptance** | No | No | No | Yes | Yes |
| **Egan (# violations; which)** | 1 (WlogP) | 1 (WlogP) | 1 (WlogP) | 0 | 0 |
| **Egan Acceptance** | No | No | No | Yes | Yes |
| **Muegge (# violations; which)** | 1 (XlogP) | 3 (MW; XlogP; #Rotatable Bonds) | 3 (MW; XlogP; #Rotatable Bonds) | 2 (MW; #Carbons) | 2 (MW; #Carbons) |
| **Muegge Acceptance** | No | No | No | No | No |
| **Leadlikeness (# violations; which)** | 3 (MW; XlogP; #Rotatable Bonds) | 3 (MW; XlogP; #Rotatable Bonds) | 3 (MW; XlogP; #Rotatable Bonds) | 1 (MW) | 1 (MW) |
| **Leadlikeness Acceptance** | No | No | No | No | No |
| **Bioavailability score** | 0.55 | --- | --- | 0.85 | 0.55 |
| **Synthetic accessibility score (SAscore)** | 3.78[a] / 5.17[bc] | 4.58 | 4.71 | 1[a] / 1.74[bc] | 1[a] / 3.42[b] |
| **Natural product-likeness score (Npscore)** | 1.50 | 0.81 | 0.80 | --- | -0.20 |
| **Fsp3** | 0.79 | 0.73 | 0.70 | 0.75 | 0.75 |
| **Brenk (Structural alert)** | 0 | 0 | 0 | 0 | 0 |
| **Medicinal Chemistry Evaluation (MCE-18)** | 56.08 | 9 | 9 | 0 | 0 |
| **PAINS (Pan Assay Interference compounds)** | 0 | 0 | 0 | 0 | 0 |
| **Alarm NMR Rule (Thiol reactive compounds)** | 1 | 0 | 0 | 0 | 0 |
| **BMS Rule (reactive compounds)** | 1 | 1 | 1 | 0 | 0 |
| **Chelator Rule (chelating compounds)** | 0 | 0 | 0 | 0 | 0 |
| **QED (Desirability)** | 0.36 | 0.02 | 0.02 | 0.54 | 0.46 |

a - http://www.swissadme.ch/; b - https://admetmesh.scbdd.com/; c - https://bidd.group/NPASS/index.php

***Table S5.*** Target prediction for Ergosterol, Octacosanol and N-Palmitoylshingomyelin.

| Target Prediction | | Ergosterol | | Octacosanol | | N-Palmitoylsphingomyelin | |
|---|---|---|---|---|---|---|---|
| | | **SwissTargetPrediction** | **SEA** | **SwissTargetPrediction** | **SEA** | **SwissTargetPrediction** | **SEA** |
| **Target_1** | **Name** | **Vitamin D receptor (VDR)** | **Delta(24)-sterol redutase (DHCR24)** | Transient receptor potential cation channel subfamily M member 8 | Dynamin-1 (DNM1) | Lysophosphatidic acid receptor Edg-7 (LPAR3) | Lysophosphatidic acid receptor Edg-7 (LPAR3) |
| | **Uniprot ID** | **P11473** | **Q15392** | Q7Z2W7 | Q05193 | Q9UBY5 | Q9UBY5 |
| | **Activity** | **Nuclear receptor of calcitriol, the active form of vitamin D3** | **Catalyzes cholesterol biosynthesis; can protect cells from oxidative stress** | Receptor-activated non-selective cation channel involved in detection of sensations such as coolness (cold temperature) | Involved in vesicular trafficking processes and in receptor-mediated endocytosis | Receptor for lysophosphatidic acid, a mediator of diverse cellular activities. May play a role in the development of ovarian cancer | Receptor for lysophosphatidic acid, a mediator of diverse cellular activities. May play a role in the development of ovarian cancer |
| **Target_2** | **Name** | **Protein-tyrosine phosphatase 1B (PTPN1)** | Steroid 17-alpha-hydroxylase/17,20lyase (CYP17A1) | Carbonic anhydrase II (CA2) | **All-trans-retinol dehydrogenase [NAD(+)]ADH7 (ADH7)** | Phospholipase A2, membrane associated (PLA2G2A) | Lysophosphatidic acid receptor 4 (LPAR4) |
| | **Uniprot ID** | **P18031** | P05093 | P00918 | **P40394** | P14555 | Q99677 |
| | **Activity** | **Acts as a regulator of endoplasmic reticulum unfolded protein response** | Involved in corticoid and androgenbiosynthesis | Catalyses the reversible hydration of carbon dioxide | **Catalyzes the NAD-dependent oxidation of all-trans-retinol, alcohol and omega-hydroxy fatty acids and their derivatives** | Secretory calcium-dependent phospholipase A2 that primarily targets extracellular phospholipids with implications in host antimicrobial defense, inflammatory response and tissue regeneration. | Receptor of LPA, a mediator of diverse cellular activities |
| **Target_3** | **Name** | Glycine receptor subunit alpha-1 (GLRA1) | **NPC1-like intracellular cholesterol transporter 1 (NPC1L1)** | Carbonic anhydrase I (CA1) | Geranylgeranyl pyrophosphate synthase (GGPS1) | Phospholipase A2 group 1B (PLA2G1B) | Toll-like receptor 2 (TLR2) |
| | **Uniprot ID** | P23415 | **Q9UHC9** | P00915 | O95749 | P04054 | O60603 |
| | **Activity** | Play a major role in mediating fast inhibitory neurotransmission in the spinal cord and brain stem | **Is the direct molecular target of ezetimibe, a drug that inhibits cholesterol absorption and is approved for the treatment of hypercholesterolemia** | Catalyses the reversible hydration of carbon dioxide | Catalyzes the trans-addition of the three molecules of IPP onto DMAPP to form geranylgeranyl pyrophosphate, an important precursor of carotenoids and geranylated proteins. | Secretory calcium-dependent phospholipase A2 that primarily targets dietary phospholipids in the intestinal tract | Cooperates with LY96 to mediate the innate immune response to bacterial lipoproteins and other microbial cell wall components |

***Table S6.*** Target prediction $\alpha$-Tocopherol, Phosphatidylcholines (16:0/20:5) and (16:0/22:6), Butyrate and Sodium Butyrate.

| Target Prediction | | $\alpha$-Tocopherol (Vitamin E) | | Phosphatidylcholines (16:0/20:5) and (16:0/22:6) | | Butyrate | | Sodium Butyrate |
|---|---|---|---|---|---|---|---|---|
| | | **SwissTargetPrediction** | **SEA** | **SwissTargetPrediction** | **SEA** | **SwissTargetPrediction** | **SEA** | **SEA** |
| **Target_1** | **Name** | PH domain leucine-rich repeat-containing protein phosphatase 1 (PHLPP1) | **Alpha-tocopherol transfer protein (TTPA)** | P-selectin (SELP) | Lysophosphatidic acid receptor 4 (LPAR4) | **Histone deacetylase 3 (HDAC 3)** | Gamma-butyrobetaine dioxygenase (BBOX1) | Cocaine Esterase (CES2) |
| | **Uniprot ID** | O60346 | **P49638** | P16109 | Q99677 | **O15379** | O75936 | O00748 |
| | **Activity** | Inhibits cancer cell proliferation and may act as a tumor supressor | **Binds alpha-tocopherol, enhances its transfer between separate membranes and stimulates its release from liver cells** | Mediates rapid rolling of leukocyte over vascular surfaces during the initial steps in inflammation | Receptor for lysophosphatidic acid (LPA), a mediator for diverse cellular activities | **Deacetylation of lysine residues** | Catalyzes the formation of L-carnitine from gamma-butyrobetaine | Involved in the detoxification of xenobiotics and in the activation of ester and amide prodrugs |
| **Target_2** | **Name** | Serine/threonine-protein kinase ILK-1 (ILK) | PH domain leucine-rich repeat-containing protein phosphatase 1 (PHLPP1) | Platelet activting factor receptor (PTAFR) | Lysophosphatidic acid receptor 6 (LPAR6) | Egl nine homolog 1 (EGLN1) | Histone lysine demethylase PHF8 (PHF8) | Liver carboxylesterase 1 (CES1) |
| | **Uniprot ID** | Q13418 | O60346 | P25105 | P43657 | Q9GZT9 | Q9UPP1 | P23141 |
| | **Activity** | Receptor-proximal protein kinase regulating integrin-mediated signal transduction | Inhibits cancer cell proliferation and may act as a tumor supressor | A chemotactic phospholipid mediator that possesses potent inflamatory, smooth-muscle contractile and hypotensive activity | Binds to oleoyl-L-alpha-lysophosphatidic acid (LPA); important for the maintenance of hair growth and texture | Cellular oxygen sensor | Plays a key role cell cycle progression , rDNA transcription and brain development | Involved in the detoxification of xenobiotics and in the activation of ester and amide prodrugs |
| **Target_3** | **Name** | Serine/threonine-protein kinase AKT (AKT1) | Cholinesterase (BCHE) | Autotaxin (ENPP2) | Lysophosphatidic acid receptor 2 (LPAR2) | **GABA transporter 1 (SLC6A1)** | Lysine-specific demethylase 7A (KDM7A) | Organic Anion transporter 3 (SLC22A8) |
| | **Uniprot ID** | P31749 | P06276 | Q13822 | Q9HBW0 | **P30531** | Q6ZMT4 | Q8TCC7 |
| | **Activity** | Regulate many processes including metabolism, proliferation, cell survival, growth and angiogenesis | Contributes to the inactivation of the neurotransmitter acetylcholine; can degrade neurotoxic organophosphate esters | Involved in several motility-related processess such as angiogenesis and neurite outgrowth | Receptor for lysophosphatidic acid (LPA); plays a key role in phospholipase C-beta signaling pathway | **Mediates transport of gamma-aminobutyric acid** | Required for brain development | May participate in the detoxification/ renal excretion of drugs and xenobiotics |
